# Theoretical insight into the interaction between SnX_2_ (X = H, F, Cl, Br, I) and benzene

**DOI:** 10.1007/s00894-016-3053-6

**Published:** 2016-08-15

**Authors:** Piotr Matczak

**Affiliations:** Department of Theoretical and Structural Chemistry, Faculty of Chemistry, University of Łódź, Pomorska 163/165, 90-236 Lodz, Poland

**Keywords:** Tin complexes, Divalent tin, Benzene, Intermolecular interaction

## Abstract

For a series of five model complexes composed of a singlet SnX_2_ molecule (X = H, F, Cl, Br, I) and a benzene molecule, the first-principles calculations of their energetics and the analysis of their electron density topology have been performed. The CCSD(T)/CBS interaction energy between SnX_2_ and C_6_H_6_ fall into the range between −10.0 and −11.2 kcal/mol, which indicates that the complexes are rather weakly bound. The relevant role of electrostatic and dispersion contributions to the interaction energy between SnX_2_ and C_6_H_6_ is highlighted in the results obtained from the symmetry-adapted perturbation theory (SAPT). The electron density topological analysis has been carried out using the quantum theory of atoms in molecules (QTAIM) and the noncovalent interactions (NCI) visualization index. Both QTAIM and NCI prove the closed-shell, noncovalent and attractive character of the interaction. A very small charge transfer from C_6_H_6_ to SnX_2_ has been detected. The formation of the five complexes is accompanied by the electron density deformations that are spatially restricted mostly to the region around the Sn atom and its adjacent C atom. The results presented in this work shed some light on the nature of the interactions associated with crystalline structural motifs involving low-valent tin complexed with neutral aryl rings.

**Electronic supplementary material:**

The online version of this article (doi:10.1007/s00894-016-3053-6) contains supplementary material, which is available to authorized users.

## Introduction

Interaction between metals and π-electron systems of neutral aryl rings is an interesting bonding motif for supramolecular self-assembly [1]. For instance, various transition metals interacting with benzene rings form both simple π-complexes in the gas phase [2–4] and larger supramolecular structures with this bonding motif [5–7]. However, the knowledge on the complexes of the heavier metals of groups 13–16 with neutral arenes is rather limited [8]. Tin is a good example in this context. The results of crystallographic studies [9] indicate that intermolecular Sn · · · π interactions often involve low-valent tin atoms, and therefore the complexation of stannylenes [10] by neutral aryl rings has attracted much interest in recent years [11–14].

In this work, a series of five systems composed of a singlet tin(II) dihydride or dihalide SnX_2_ (X = H, F, Cl, Br, I) molecule and a benzene molecule is considered. From a computational viewpoint, such systems may be regarded as model systems that approximate real systems in which the complexation of stannylenes by the π-electron cloud of neutral aryl rings occurs [9]. We focus mostly on the energetic and electron density topological description of the interaction between SnX_2_ and C_6_H_6_ in the resulting complexes: SnH_2_ · · · C_6_H_6_ (**1**), SnF_2_ · · · C_6_H_6_ (**2**), SnCl_2_ · · · C_6_H_6_ (**3**), SnBr_2_ · · · C_6_H_6_ (**4**), and SnI_2_ · · · C_6_H_6_ (**5**). Such quantum-chemical theories as the symmetry-adapted perturbation theory [15, 16] in both its traditional formulation (HF-SAPT) and its variant based on the density functional theory (DFT-SAPT), the quantum theory of atoms in molecules (QTAIM) [17], and the noncovalent interactions (NCI) visualization index [18] are used to provide an in-depth insight into this interaction. The present investigation is an extension of our previous work [19] in which only limited characteristics of **1**–**5** were obtained because some basic geometrical and energetic parameters were sufficient for our benchmark assessment of the accuracy of the Møller–Plesset second-order perturbation theory (MP2) and the density functional theory (DFT). There is also another theoretical study of the interaction of SnX_2_ with C_6_H_6_ [20]. In that study, the properties of various SnX_2_ molecules were characterized by conceptual DFT reactivity indices and the complexation of SnX_2_ with a series of potential aromatic π-donors was examined. In particular, it was deduced from the results of natural bond orbital (NBO) calculations that the most important orbital interaction for this complexation was the overlap of the formally empty *p*-orbital on the Sn atom and the π-orbitals of the C_6_H_6_ molecule. Here, we employ not only a wide variety of first-principles methods for inspecting the energetics of the interaction between SnX_2_ and C_6_H_6_ but additionally we explore the interaction in the complexes **1**–**5** from the perspective of their electron density topology.

## Computational details

The geometries of **1**–**5** are taken from our previous study [19] in which they were optimized at the ωB97X/aug-cc-pVTZ(-PP) level of theory [21–24]. These geometries are characterized by the absence of imaginary vibrational frequencies and they correspond to global minima on the potential energy surfaces of **1**–**5** (see also section S1 in Electronic Supplementary Material). Throughout this entire work, the aug-cc-pVTZ basis set [22] is ascribed to the atoms of H, C, F, Cl, and Br, whereas the aug-cc-pVTZ-PP basis set [23] is used for Sn and I. Their 28 core electrons are described by the corresponding energy-consistent Stuttgart/Cologne MDF pseudopotentials [24]. These pseudopotentials allow us to indirectly account for relativistic effects in Sn and I.

The formation of each complex is characterized by its complexation energy *E*_complex_ that is defined as

1 $$ {E}_{\mathrm{complex}}={E}_{def}+{E}_{int} + \varDelta ZPVE $$

where *E*_def_ is the so-called deformation energy needed to change the geometries of SnX_2_ and C_6_H_6_ from those exhibited by isolated molecules to those observed in the complex, *E*_int_ is the interaction energy between SnX_2_ and C_6_H_6_ in the complex and Δ*ZPVE* denotes the difference in the unscaled zero-point vibrational energies of the isolated SnX_2_ and C_6_H_6_ molecules and the SnX_2_ · · · C_6_H_6_ complex. *E*_int_ can be computed using either supermolecular or perturbative approach. According to the former, subtracting the total energies of SnX_2_ and C_6_H_6_ in their geometries observed in the complex from the total energy of the SnX_2_ · · · C_6_H_6_ complex constitutes *E*_int_. In this work, both DFT methods (such as SVWN [25, 26], BLYP [27, 28], B3LYP [28, 29], M06-2X [30] and ωB97X [21]), and wave function theory (WFT) methods (such as HF [31, 32], MP2 [33], SCS-MP2 [34], CCSD [35], and CCSD(T) [35]) are used to obtain *E*_int_ within the framework of the supermolecular approach. The *E*_int_ energies yielded by the HF and DFT methods are corrected for the basis set superposition error using the full counterpoise method of Boys and Bernardi [36]. The “half-half” counterpoise correction [37] is included in the *E*_int_ energies calculated using MP2, SCS-MP2, CCSD, and CCSD(T). The *E*_int_ energies at the CCSD(T) level of theory are additionally extrapolated to the complete basis set (CBS) limit via a composite scheme (for details, see section S2). The MP2, SCS-MP2, CCSD, and CCSD(T) methods make use of the frozen core approximation in the treatment of core electrons. All the calculations described in this paragraph have been done with Gaussian D.01 [38] and TURBOMOLE 6.6 [39] (for the DFT and WFT methods, respectively).

The SAPT method is used to determine the *E*_int_ energy between SnX_2_ and C_6_H_6_ in a perturbative manner. Within the framework of this method, *E*_int_ is expressed as a sum of several energy terms that can be grouped into four principal components with clear physical meanings. The components covering exchange (*E*_exch_), electrostatics (*E*_elst_), induction (*E*_ind_), and dispersion (*E*_disp_) are assumed to include the following energy terms occurring in the SAPT expansion of *E*_int_:

2 $$ {E}_{\mathrm{exch}} = {E}_{\mathrm{exch}}^{(1)} $$

3 $$ {E}_{\mathrm{elst}} = {E}_{\mathrm{elst}}^{(1)} $$

4 $$ {E}_{ind}={E}_{\mathrm{ind}}^{(2)}+{E}_{\mathrm{exch}-\mathrm{i}\mathrm{n}\mathrm{d}}^{(2)} + \delta {E}_{HF} $$

5 $$ {E}_{\mathrm{disp}}={E}_{\mathrm{disp}}^{(2)}+{E}_{\mathrm{exch}-\mathrm{disp}}^{(2)} $$

The HF-SAPT [15, 16] and DFT-SAPT [40, 41] variants are employed. The gradient-regulated asymptotic correction of Grüning et al. [42] with the PBE0 functional [43] is introduced into the DFT-SAPT calculations of *E*_int_ in order to improve the asymptotic behavior of the DFT-SAPT variant. All the SAPT calculations have been performed using the MOLPRO 2012.1 program [44, 45].

The QTAIM analysis of the topology of the electron density in **1**–**5** and the calculations of QTAIM properties have been done with the AIMAll 14.11.23 program [46]. The Multiwfn 3.3 program [47] has been used to perform the NCI analysis and to obtain a signature of electron pair distribution in terms of the electron localization function (ELF) [48]. The results are visualized using Jmol 14.2 [49], AIMStudio 14.11.23 [46], and VMD 1.9 [50].

## Results and discussion

### Structure and stability

**Fig. 1:**
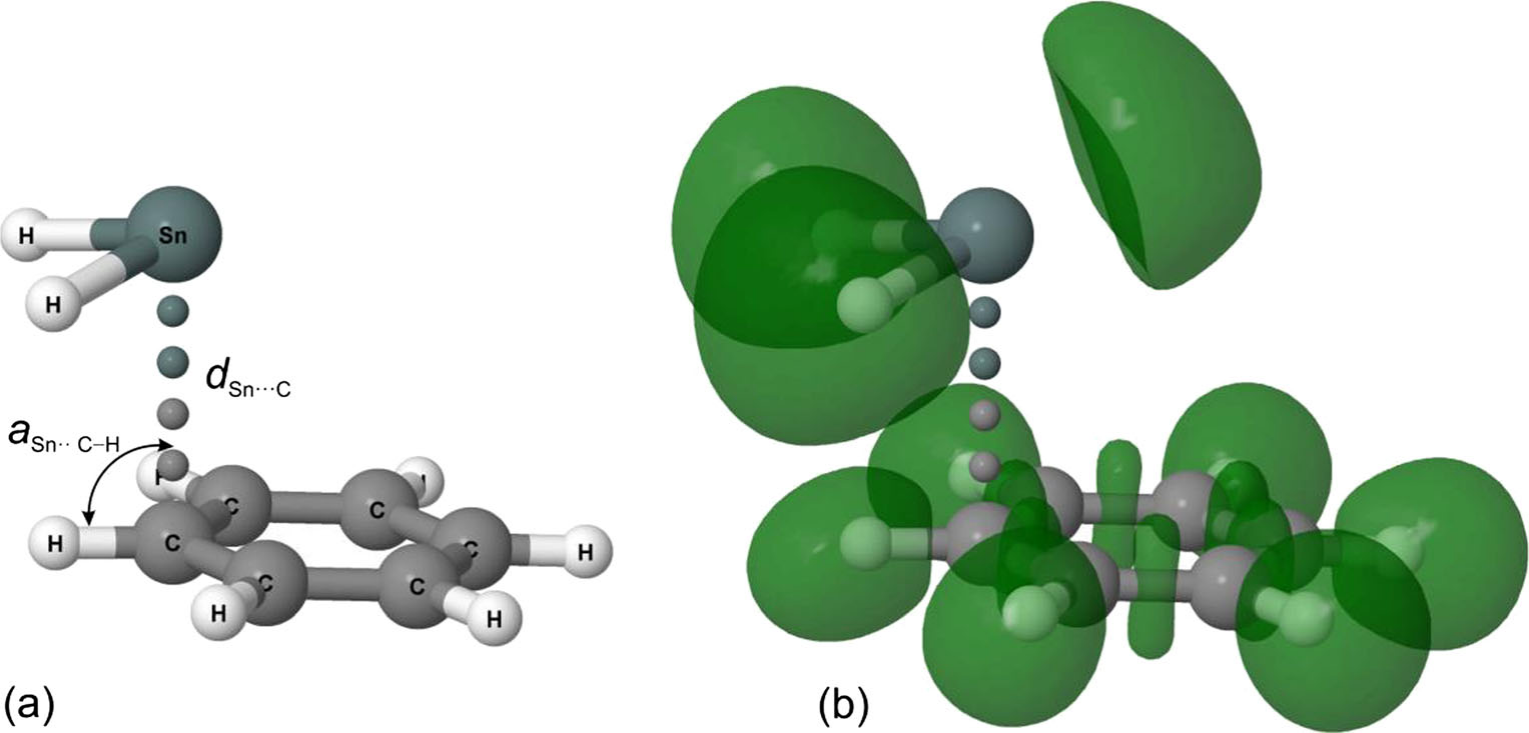
Optimized geometry of **1** with two characteristic geometrical parameters marked (**a**) or ELF isosurfaces shown (**b**). These isosurfaces are plotted with a contour value of 0.85

Let us start by presenting the optimized geometries of **1**–**5**. All these complexes exhibit a marked structural similarity in which the SnX_2_ molecule is arranged above the C_6_H_6_ ring (see Fig. 1a). The molecular plane of SnX_2_ is approximately parallel to the plane of the C_6_H_6_ ring. All five complexes are of *C*_*s*_ symmetry. The Sn atom sits nearly on one of the C atoms, and the X atoms are directed outward the C_6_H_6_ ring. The distance between Sn and the C atom beneath (*d*_Sn···C_) falls in a narrow range between 2.919 and 3.076 Å (see Table S6 in Electronic Supplementary Material). This is much smaller than the sum of the Sn- and C-atom van der Waals radii (2.42 Å for Sn and 1.77 Å for C [51]). The values of the *a*_Sn···C–H_ angle do not differ significantly from 90°, and therefore, they indicate the *η*^1^ type of complexation for **1**–**5**.

The aforementioned, almost parallel orientation of the SnX_2_ and C_6_H_6_ molecular planes means that the formally empty *p*-orbital of the Sn atom is nearly perpendicular to the plane of the C_6_H_6_ ring. In such an orientation, the π-cloud overlaps the empty *p*-orbital effectively [20]. The lone electron pair of the Sn atom is positioned opposite the X atoms. Its localization is confirmed by the corresponding ELF isosurface depicted in Fig. 1b.

The comparison of the isolated SnX_2_ and C_6_H_6_ molecules with the corresponding molecular fragments of **1**–**5** reveals that SnX_2_ and C_6_H_6_ do not suffer any significant structural variations upon complexation (see Table S6). A minor effect of complexation on the geometries of SnF_2_ and C_6_H_6_ molecular fragments constituting a 1:1 complex was confirmed experimentally, using matrix IR spectroscopy [52]. The vibrational frequencies measured for this complex were close to those of SnF_2_ and benzene substrates, and on this basis, only a slight distortion of SnF_2_ and C_6_H_6_ geometries was deduced. The complexation of Sn(II) by a six-membered aromatic ring was also detected in several crystal structures [11–14]. The structures often revealed the *η*^6^ type of Sn(II) complexation, with the distances between Sn(II) and the centroid of the interacting aromatic ring within the range from 3.2 to 4 Å [11, 12]. The lower end of this range is slightly larger than the *d*_Sn···C_ values found for **1**–**5**. Such a shortening of the distance between Sn(II) and the molecular plane of aromatic ring is a consequence of the *η*^1^ type of complexation in **1**–**5**.

Energetic effects associated with the complexation of SnX_2_ with C_6_H_6_ are summarized in Table 1. As evidenced by the values of *E*_complex_, the formation of **1**–**5** from the isolated SnX_2_ and C_6_H_6_ molecules is energetically favorable (that is, *E*_complex_ < 0), although the resulting stabilization of the complexes does not exceed several kcal/mol. The complexation of SnH_2_ turns out to be slightly less energetically favorable than the complexation of tin(II) dihalides. The values of *E*_def_ are small, which is a consequence of the minor structural reorganization of SnX_2_ and C_6_H_6_ upon complexation. For all five complexes, the interaction energy *E*_int_ contributes most to *E*_complex_. The values of *E*_int_ fall in a narrow range between −9.1 and −9.7 kcal/mol, which obviously suggests that the interaction between SnX_2_ and C_6_H_6_ in **1**–**5** is in fact quite similar. The difference in the energetics of the complexes becomes more significant with the Δ*ZPVE* component included.

**Table 1:** Complexation energies and their components calculated using the ωB97X method

Energy	Complex				
	**1**	**2**	**3**	**4**	**5**
*E*_def_	0.1	0.2	0.3	0.4	0.3
*E*_int_	−9.3	−9.7	−9.7	−9.5	−9.1
Δ*ZPVE*	1.6	0.7	0.6	0.6	0.6
*E*_complex_	−7.6	−8.8	−8.8	−8.5	−8.2

All values in kcal/mol

The *E*_int_ values shown in Table 1 indicate that the interaction between SnX_2_ and C_6_H_6_ can be classified into the category of weak intermolecular interactions. The interaction investigated here turns out to be much weaker than those involving benzene and alkali or alkaline-earth metal cations [53, 54] but stronger than the interactions in SF_2_ · · · C_6_H_6_ [55] and SeY_2_ · · · C_6_H_6_ (Y = H, F, Cl) [56].

The interaction energies of **1**–**5** have also been computed at various DFT and WFT levels, all using the geometries optimized by ωB97X/aug-cc-pVTZ(-PP). The calculated values of *E*_int_ are reported in Table 2 (the *E*_int_ values that are uncorrected by the counterpoise method can be found in Table S7). We now test the reliability of the *E*_int_ energies obtained from less computationally expensive levels of theory against the CCSD(T)/CBS results.

**Table 2:** Interaction energies calculated using various methods

Method	Complex				
	**1**	**2**	**3**	**4**	**5**
HF	−2.1	−4.5	−2.9	−2.6	−2.1
SVWN	−14.5	−13.3	−13.1	−12.8	−12.2
BLYP	−3.3	−1.9	−0.9	−0.4	0.3
BLYP-D3(BJ)	−11.4	−10.8	−11.5	−11.6	−11.5
B3LYP	−4.6	−4.3	−3.2	−2.9	−2.2
B3LYP-D3(BJ)	−11.3	−11.7	−12.2	−12.2	−12.0
M06-2X	−10.4	−11.7	−11.1	−10.9	−10.8
ωB97X	−9.3	−9.7	−9.7	−9.5	−9.1
HF-SAPT	−11.6	−13.6	−13.7	−13.8	−13.6
DFT-SAPT	−8.8	−10.0	−10.4	−10.5	−10.3
MP2	−13.0	−13.1	−14.5	−14.6	−15.5
SCS-MP2	−10.5	−11.0	−11.9	−11.9	−12.5
CCSD	−9.2	−10.6	−10.4	−10.2	−10.7
CCSD(T)	−10.7	−11.6	−11.8	−11.7	−12.1
CCSD(T)/CBS	−10.0	−10.9	−11.2	−11.1	−11.1

All values in kcal/mol

The HF method predicts that the interaction between SnX_2_ and C_6_H_6_ leads to the stabilization of **1**–**5** but the resulting *E*_int_ values are very small. Thus, the omission of electron correlation energy results in a major underestimation of the strength of the interaction between SnX_2_ and C_6_H_6_.

The DFT methods present diverse performance, depending on the generation of a given DFT method. The SVWN functional leads to a typical overbinding [57]. In contrast to SVWN, the BLYP and B3LYP functionals predict that the complexes **1**–**5** are bound too weakly or even unbound (BLYP produces *E*_int_ > 0 for **5**). The *E*_int_ values obtained from B3LYP differ slightly from the corresponding B3LYP values reported in Ref. [20]. These differences originate from a greater number of core electrons described by the pseudopotential of Sn atom during the calculations carried out in Ref. [20]. A common method that can improve the performance of BLYP and B3LYP in predicting the interaction energy of noncovalent complexes is to include an empirical dispersion correction. Here, Grimme’s D3 term with Becke-Johnson damping (D3(BJ)) [58] is employed for BLYP and B3LYP. The application of the D3(BJ) term allows these two functionals to overcome the underestimation of *E*_int_ for **1**–**5**. However, both BLYP-D3(BJ) and B3LYP-D3(BJ) yield a minor overbinding in **1**–**5**, which suggests that the D3(BJ) term generally tends to overestimate slightly the role of long-range electron correlation in these complexes. This extends findings reported in a prior study [59]. It was shown therein that B3LYP combined with Grimme’s dispersion correction yielded overbinding for hydrogen-bonded and ionic complexes. A slight overestimation of hydrogen-bond energy in DNA base pairs was also detected for the combination of BLYP with Grimme’s dispersion correction [60, 61]. More recent DFT generations, represented in Table 2 by M06-2X and ωB97X, produce the *E*_int_ energies that mirror the CCSD(T)/CBS results fairly closely. It is known that the ωB97X functional has proved to be highly successful at providing accurate bond energies for compounds containing transition metals [62], as well as interaction energies for complexes of Sn(II) [19] and Sn(IV) [63, 64]. The good performance of M06-2X in predicting the *E*_int_ energies of **1**–**5** seems to confirm the important role of medium-range electron correlation for these complexes [30, 65].

As for the SAPT method, its HF-SAPT variant overestimates the strength of the interaction between SnX_2_ and C_6_H_6_, while the DFT-SAPT variant demonstrates the opposite tendency. However, the former produces the *E*_int_ energies with worse accuracy relative to the CCSD(T)/CBS results than the latter does. It is so because the HF-SAPT variant applied here makes use of the simplest SAPT formulation, usually called HF-SAPT0 [66], that neglects the effects of intramonomer correlation. The intramonomer correlation effects are accounted for within the framework of the DFT-SAPT variant. It has been recently reported that DFT-SAPT generally underbinds H-bonded complexes [67] and it also proves to be the case with the interaction between SnX_2_ and C_6_H_6_. The maximum DFT-SAPT underestimation of the strength of the interaction in **1**–**5** amounts to 1.2 kcal/mol — this is observed for **1**.

The performance of the advanced WFT methods also demonstrates some characteristic features. The MP2 method systematically overestimates the strength of the interaction between SnX_2_ and C_6_H_6_ due to the omission of the repulsive intramolecular correlation correction within this method [68, 69]. The inclusion of Grimme’s spin-component scaling (SCS) scheme [34] in the MP2 correlation energy reduces the overbinding of **1**–**5** but the *E*_int_ value of **5** is still overestimated by 1.4 kcal/mol. The overestimation occurring in SCS-MP2 results from the application of the “half-half” correction of the basis-set superposition error. However, this correction leads to better agreement with the CCSD(T)/CBS interaction energies than the use of either uncorrected or full counterpoise-corrected values (see Table S8). Similarly to SCS-MP2, the CCSD(T) method also shows an overbinding tendency in *E*_int_. Again, it is due to the application of the “half-half” correction. The *E*_int_ values yielded by CCSD are too small, which points at the importance of triple excitations in the correlation energy of **1**–**5**.

The CCSD(T)/CBS method, which is deemed to give the most accurate estimates of *E*_int_ in **1**–**5**, confirms that the interaction between SnX_2_ and C_6_H_6_ should be classified as weak, with the *E*_int_ values between −10.0 and −11.2 kcal/mol. Comparing the CCSD(T)/CBS results with the ωB97X ones, we see that the former indicate a more diversified strength of the interaction in the investigated series of complexes. The SnCl_2_ molecule is bound strongest to the C_6_H_6_ molecule, whereas the bonding of SnH_2_ to C_6_H_6_ turns out to be particularly weak. For the complexes containing the tin(II) dihalides, no monotonous regularity in their CCSD(T)/CBS *E*_int_ values is found while X becomes heavier and heavier.

### Interaction energy decomposition

The next step of this work is to characterize the physical nature of the interaction between SnX_2_ and C_6_H_6_ using the SAPT method. We first focus on the DFT-SAPT interaction energy terms calculated for **1**–**5** and listed in Table 3. For all five complexes their electrostatic first-order term *E*_elst_^(1)^ is negative, which can be naively understood as a result of the attraction between the π-cloud and the positively-charged Sn atom (its QTAIM charge ranges from 0.86 to 1.49 au in **1**–**5**). The absolute values of *E*_elst_^(1)^ are always noticeably smaller than the corresponding values of the first-order exchange term *E*_exch_^(1)^. The magnitude of the second-order induction *E*_ind_^(2)^ (SnX_2_ → C_6_H_6_) is several times larger than its *E*_ind_^(2)^ (SnX_2_ ← C_6_H_6_) analog. It means that the C_6_H_6_ molecule is easily polarized by the SnX_2_ molecule, whereas the polarization in the opposite direction is significantly less pronounced. The *E*_ind_^(2)^(SnX_2_ ← C_6_H_6_) term of each complex is practically counterbalanced by the corresponding *E*_exch ‐ ind_^(2)^ (SnX_2_ ← C_6_H_6_) term, while *E*_ind_^(2)^(SnX_2_ → C_6_H_6_) is quenched up to ca. 66 % by its exchange counterpart. An even smaller percentage quenching of *E*_disp_^(2)^ is attributed to *E*_exch ‐ disp_^(2)^. The *δE*_HF_ term destabilizes all five complexes. This term collectively gathers mostly third- and higher-order induction and exchange-induction contributions. The values of *δE*_HF_ decrease gradually in the sequence from **1** to **5**, that is, with growing *d*_Sn···C_ distance. This indicates that the higher-order effects become increasingly important at shorter distances. Even though the inclusion of *δE*_HF_ into the DFT-SAPT *E*_int_ values leads to the underestimation of the strength of the interaction in **1**–**5**, the presence of *δE*_HF_ is more beneficial for the accuracy of *E*_int_ than omitting this term. This is a well-documented feature of SAPT computations involving pseudopotentials [70].

**Table 3:** SAPT interaction energy terms calculated using the DFT-SAPT variant

Energy term	Complex				
	**1**	**2**	**3**	**4**	**5**
*E*_exch_^(1)^	17.2	19.0	20.4	20.5	20.4
*E*_elst_^(1)^	−12.0	−14.0	−14.1	−14.0	−13.7
*E*_ind_^(2)^(SnX_2_ → C_6_H_6_)	−31.7	−28.1	−25.8	−24.6	−22.4
*E*_exch ‐ ind_^(2)^(SnX_2_ → C_6_H_6_)	21.1	18.5	17.0	16.3	14.5
*E*_ind_^(2)^(SnX_2_ ← C_6_H_6_)	−4.9	−6.0	−7.2	−7.8	−8.7
*E*_exch ‐ ind_^(2)^(SnX_2_ ← C_6_H_6_)	4.7	5.8	7.0	7.6	8.6
*E*_disp_^(2)^	−11.5	−11.6	−13.4	−13.9	−14.4
*E*_exch ‐ disp_^(2)^	2.3	2.4	2.7	2.8	3.0
*δE*_HF_	6.0	4.1	3.0	2.5	2.4

All values in kcal/mol

Next, let us examine the relative importance of four principal components of *E*_int_ in order to establish the physical origin of the interaction between SnX_2_ and C_6_H_6_. Table 4 summarizes the results of grouping either HF-SAPT or DFT-SAPT interaction energy terms into four principal components. We now take a closer look at the DFT-SAPT components but the findings we make using these components are also valid for the HF-SAPT components.

**Table 4:** Four principal components of SAPT interaction energies calculated using the HF-SAPT and DFT-SAPT variants

Principal component	Complex				
	**1**	**2**	**3**	**4**	**5**
*E*_exch_^HF ‐ SAPT^	18.7	19.3	21.2	21.3	21.1
*E*_elst_^HF ‐ SAPT^	−14.0 (46.2)	−16.1 (48.8)	−16.2 (46.4)	−16.0 (45.7)	−15.7 (45.1)
*E*_ind_^HF ‐ SAPT^	−6.8 (22.6)	−7.7 (23.5)	−7.9 (22.6)	−7.9 (22.4)	−7.5 (21.6)
*E*_disp_^HF ‐ SAPT^	−9.5 (31.2)	−9.1 (27.7)	−10.8 (31.0)	−11.2 (31.8)	−11.6 (33.3)
*E*_exch_^DFT ‐ SAPT^	17.2	19.0	20.4	20.5	20.4
*E*_elst_^DFT ‐ SAPT^	−12.0 (46.1)	−14.0 (48.4)	−14.1 (45.9)	−14.0 (45.1)	−13.7 (44.6)
*E*_ind_^DFT ‐ SAPT^	−4.8 (18.3)	−5.7 (19.8)	−6.0 (19.4)	−6.0 (19.3)	−5.6 (18.3)
*E*_disp_^DFT ‐ SAPT^	−9.3 (35.6)	−9.2 (31.8)	−10.7 (34.7)	−11.0 (35.6)	−11.4 (37.1)

The percentage share of individual attractive components in the total attraction between SnX_2_ and C_6_H_6_ is given in parentheses

Energy components in kcal/mol

For all five complexes, the DFT-SAPT decomposition of their *E*_int_ energies yields three attractive components, namely *E*_elst_^DFT ‐ SAPT^, *E*_ind_^DFT ‐ SAPT^ and *E*_disp_^DFT ‐ SAPT^. These three components provide sufficient stabilization to overcome the repulsive exchange component, and therefore, the resultant *E*_int_ values are negative (see Table 2). Among the attractive components, electrostatics represents the most energetically favorable contribution, followed by dispersion and then by induction. The *E*_elst_^DFT ‐ SAPT^ component provides slightly less than half of the total attraction between SnX_2_ and C_6_H_6_. However, this component does not surpass the *E*_exch_^DFT ‐ SAPT^ one, and in consequence, the total contribution from the first-order DFT-SAPT energy terms remains repulsive. Therefore, the stability of **1**–**5** is due to the second- and higher-order DFT-SAPT energy terms, that are included in the *E*_ind_^DFT ‐ SAPT^ and *E*_disp_^DFT ‐ SAPT^ components. Of the two components, the latter plays the more important role. In relative terms, dispersion accounts for roughly one third of the total attraction between SnX_2_ and C_6_H_6_. The relevant role of the dispersion energy often occurs for complexes containing large, diffuse electron clouds, such as the π-electron system of benzene in our case. The stabilization arising mainly from the combined effect of electrostatics and dispersion has recently been detected also for some other benzene complexes, e.g. with HCN [71].

The absolute values of *E*_ind_^DFT ‐ SAPT^ essentially increase with the growing atomic number of the X atoms. However, there is a noticeable decrease of the magnitude of *E*_ind_^DFT ‐ SAPT^ when one goes from **4** to **5**. This results from the usage of a pseudopotential for the core electrons of the I atoms. For **2**–**5**, the magnitude of *E*_disp_^DFT ‐ SAPT^ and simultaneously the relative importance of this component increase while the halogen atoms get heavier. This is consistent with the growing polarizability of tin(II) dihalides [72]. In contrast to *E*_disp_^DFT ‐ SAPT^, the other two attractive components of *E*_int_ show a diminishing relative importance while moving throughout the series from **2** to **5**.

### Topological analysis of electron density

**Fig. 2:**
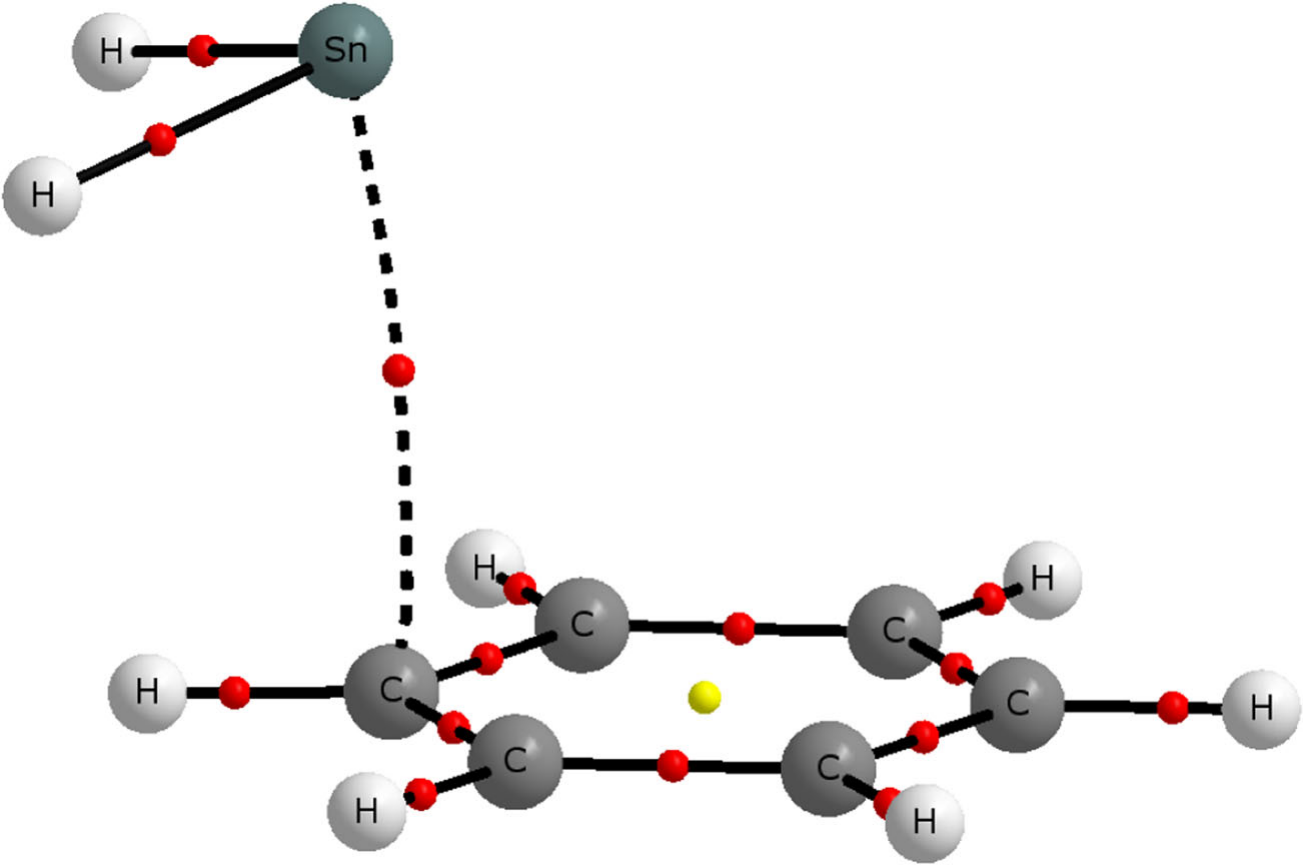
Molecular graph for **1**. BCPs are denoted by small *red circles*, while the only ring critical point is marked by a small *yellow circle*. All atoms are colored the same as in Fig. 1

Complementary information about the interaction between SnX_2_ and C_6_H_6_ can be obtained from the QTAIM topological analysis of the electron density *ρ* in **1**–**5**. The molecular graph determined for **1** is shown in Fig. 2. It is evident from the graph that there is a single bond path (BP) linking the Sn atom and the adjacent C atom of the C_6_H_6_ molecule. Such a feature of the topology of *ρ* is common to all five complexes **1**–**5**. This suggests that the formation of a Sn · · · C contact between the Sn atom and its nearest neighboring C atom of C_6_H_6_ is associated with the existence of a bonding interaction between these atoms [73]. The existence of the BP linking the Sn atom and its nearest neighboring C atom also indicates that, from the perspective of the QTAIM, the complexes **1**–**5** should be described as *η*^1^ complexes.

The topological properties of *ρ* at the bond critical point (BCP) on the BP of the Sn · · · C contact in **1**–**5** provide a more detailed QTAIM characteristics of the interaction between SnX_2_ and C_6_H_6_. The values of several such properties are collected in Table 5. The values of *ρ* decrease with growing *d*_Sn···C_ distance. The relationship between *ρ* and *d*_Sn···C_ exhibits a good linear reverse correlation, with the respective coefficient of determination R^2^ being equal to 0.96. It is due to the fact that the range of the *d*_Sn···C_ distances found for **1**–**5** is very narrow. For a broader range of distances a non-linear relationship should be rather expected, as it was demonstrated for other noncovalent interactions [74, 75]. The values of *ρ* at the BCP of the Sn · · · C contact in **1**–**5** are much smaller than typical *ρ* values at the BCP of a covalent Sn-C bond (e.g. 0.099 and 0.106 au for the Sn-C bonds of Sn(CH_3_)_2_ and Sn(CH_3_)_4_, respectively). Furthermore, the *ρ* values in Table 5 are smaller than those found for Sn · · · C contacts in the solid-state structure of dicationic tin-toluene complex [Sn(C_7_H_8_)_3_]^2+^ [13]. It is accompanied by an elongation of the Sn · · · C contact in **1**–**5** compared to the contacts of [Sn(C_7_H_8_)_3_]^2+^. The values of the Laplacian of the electron density ∇^2^*ρ* in **1**–**5** are positive, which means that the charge density is locally depleted at the BCP relative to the neighboring points in space and, in consequence, it is locally concentrated in the basins of the Sn and C atoms of the contact. The total energy density *H*, which is the sum of the electron kinetic energy density *G* and the electron potential energy density *V*, adopts the negative sign but the values of *H* are very close to zero. This may suggest that there is only a minor covalent factor contributing to the nature of the interaction between SnX_2_ and C_6_H_6_. Moreover, the low values of *ρ* and ∇^2^*ρ* > 0, together with –*V*/*G* > 1 and –*λ*_1_/*λ*_3_ < < 1, provide evidence for the lack of any appreciable covalency [17, 74]. The aforementioned criteria indicate that this interaction should be classed as the closed-shell, noncovalent interaction [17, 74]. The –*λ*_1_/*λ*_3_ criterion of the nature of interaction is calculated using the lowest *λ*_1_ and highest *λ*_3_ eigenvalues of the Hessian matrix of *ρ*. The QTAIM characteristics of the Sn · · · C bonding interaction in **1**–**5** is essentially similar to that found previously for metal-ligand bonds in some complexes of Mn [76, 77] and Zn [78].

**Table 5:** QTAIM properties at the BCP on the BP linking the Sn atom and the adjacent C atom in each of the complexes **1**–**5**

Property	Complex				
	**1**	**2**	**3**	**4**	**5**
*ρ* · 10^2^	1.776	1.688	1.660	1.617	1.557
∇^2^ *ρ* · 10^2^	3.946	3.425	3.272	3.195	3.092
*G* · 10^3^	10.330	8.828	8.426	8.147	7.777
*V* · 10^3^	−10.795	−9.094	−8.674	−8.306	−7.825
*H* · 10^4^	−4.648	−2.661	−2.475	−1.587	−0.479
–*V*/*G*	1.045	1.030	1.029	1.019	1.006
–*λ*_1_/*λ*_3_	0.185	0.228	0.227	0.225	0.221

All values in au, except for the –*V*/*G* and –*λ*_1_/*λ*_3_ ratios that are dimensionless

**Fig. 3:**
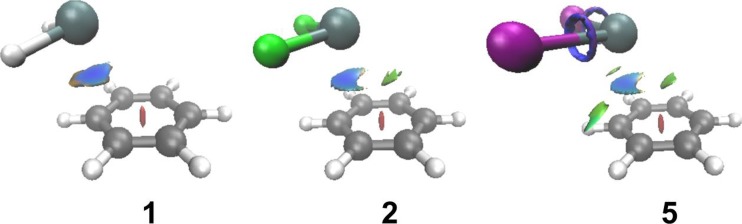
NCI isosurfaces for **1**, **2** and **5**. The isosurfaces are plotted with a reduced density gradient value of 0.35 au and they are colored from blue to red according to sign(*λ*_2_)*ρ* ranging from −0.02 to 0.02 au. The colors denoting the H, C and Sn atoms are the same as in Fig. 1. The atoms of F and I are drawn in green and violet, respectively

The standard QTAIM characterization presented above can be supplemented by the analysis of the NCI visualization index. The plots showing the NCI isosurfaces detected for **1**, **2** and **5** are presented in Fig. 3. As can be seen in these plots, the interaction between SnX_2_ and C_6_H_6_ in the complexes is characterized by a blue isosurface located between the Sn atom and its nearest neighboring C atom. The region delineated by this isosurface illustrates the occurrence of the attractive interaction between SnX_2_ and C_6_H_6_. For the complexes containing the tin(II) dihalides, additional isosurfaces representing the interaction between SnX_2_ and C_6_H_6_ appear. Such isosurfaces are located between the Sn atom and the more distant C atoms of C_6_H_6_. These isosurfaces are colored in green in Fig. 3 and they are associated with the occurrence of an extremely weak, repulsive interaction. Some other NCI isosurfaces denoting the existence of a secondary attractive interaction can in turn be found between the I atoms and their nearest neighboring H atoms in **5**. One may speculate that these isosurfaces can be ascribed to the occurrence of a very weak H-bonding, although no BP has been detected between the I and H atoms, and the distance of 3.651 Å between these atoms exceeds the sum of their van der Waals radii.

### Electron density deformations and charge transfer

**Fig. 4:**
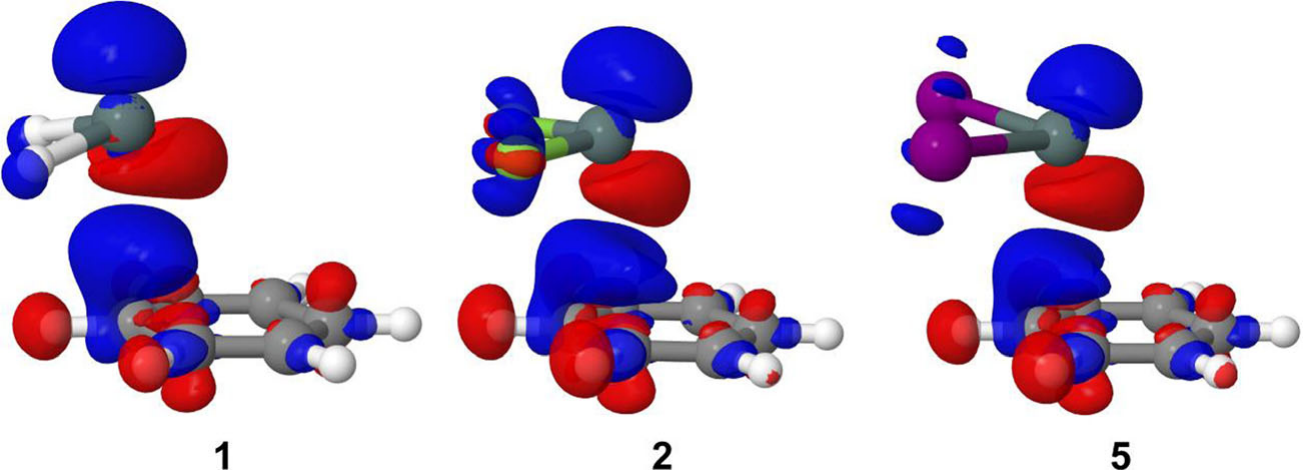
Plots of the electron density difference calculated for **1**, **2** and **5**. The blue and red isosurfaces are plotted with contour values of 0.001 and −0.001 au, respectively. The colors coding individual elements are the same as in Fig. 3

In the subsection presented above, the complexes have been examined mainly from the perspective of their *ρ* itself. Now, the comparison of this *ρ* with the electron densities of non-interacting SnX_2_ and C_6_H_6_ fragments is made in order to determine how the electron density adjusts to the interaction between SnX_2_ and C_6_H_6_. Such a comparison can conveniently be presented in the form of an electron density difference plot, as it is shown in Fig. 4 for **1**, **2** and **5**. For each of these complexes, its electron density difference has been computed as the difference between the *ρ* of the whole complex and the sum of the densities of individual SnX_2_ and C_6_H_6_ fragments in their geometries taken from the complex. The regions delineated by blue isosurfaces in Fig. 4 illustrate an increase in *ρ* arising from the interaction, while the red regions determine where *ρ* is reduced. The most relevant changes in the *ρ* distribution are detected for the Sn · · · C contact and its spatially closest neighborhood. There is a region of *ρ* reduction immediately below the Sn atom of SnX_2_, whereas an increase in *ρ* is observed above this atom. In other words, the *ρ* distribution around the Sn atom becomes polarized toward the π-electron cloud of C_6_H_6_ upon complexation. The regions of growing *ρ* are found around the X atoms and the *ρ* deformation around these atoms is enhanced while moving from X = I to X = F, that is, with the increasing electronegativity of X. The distribution of *ρ* within the C_6_H_6_ molecule also undergoes some changes upon the complexation with SnX_2_. The most prominent change can be perceived above the C atom involved in the contact with the Sn atom. The vast blue region of increased *ρ* spreads over the part of the adjacent C-H bond. This is accompanied by several regions of *ρ* loss around the H atoms on the periphery of C_6_H_6_.

An essential aspect of the interaction between SnX_2_ and C_6_H_6_ is the magnitude of the charge transfer that possibly appears as a result of complexation. The charge transfer between SnX_2_ and C_6_H_6_ is estimated here using the QTAIM atomic charges calculated for **1**–**5**. The QTAIM charges of atoms constituting the SnX_2_ fragment of the complexes are summed up, yielding the overall magnitude of charge transferred between SnX_2_ and C_6_H_6_. The magnitude of the charge transfer estimated in that manner adopts very small values, from 0.0388 au for **5** to 0.0578 au for **2**. The complexes containing the tin(II) dihalides show a gradual decrease in the magnitude of charge transfer while going throughout the series from **2** to **5**. For these complexes there is a strong linear association between the decreasing magnitude of charge transfer and the increasing value of *d*_Sn···C_ (the resulting inverse correlation between these two quantities is quantitatively characterized by R^2^ = 0.99). The charge transfer in **1** is estimated to be 0.0415 au. The formation of all five complexes leads to a small flow of electron charge from the C_6_H_6_ molecule to the SnX_2_ molecule. Thus, the SnX_2_ fragment of **1**–**5** bears a slight negative charge, from −0.0388 to −0.0578 au. The detected very small charge transfer and its direction may point at the existence of a very weak donor-acceptor π → Sn contribution to the interaction in **1**–**5**. It would be in line with the results of a previous computational study based on the NBO approach [20]. In that study, an electron donation from the π-type NBO orbitals of C_6_H_6_ to the formally empty *p*-NBO orbital on the Sn atom of SnX_2_ was indeed detected but the calculated charge transfers from C_6_H_6_ to SnX_2_ were even smaller than those reported here.

### Analysis of vibrational frequencies

The formation of **1**–**5** introduces some characteristic changes in the frequencies of vibrations occurring for the SnX_2_ and C_6_H_6_ fragments of the complexes. Table 6 presents shifts in the frequencies of three vibrations upon complex formation. The three vibrations include asymmetrical and symmetrical stretching frequencies of Sn-X bonds (*υ*_as,Sn-X_ and *υ*_s,Sn-X_) and out-of-plane deformation vibrations of C-H bonds in the C_6_H_6_ ring (*δ*_C-H_). The frequencies of these vibrations have been computed within the quantum harmonic oscillator approximation at the ωB97X/aug-cc-pVTZ(-PP) level of theory. They have not been scaled.

**Table 6:** Shifts in vibrational frequencies of **1**–**5** as the result of complex formation

Frequency shift	Complex				
	**1**	**2**	**3**	**4**	**5**
Δ*υ*_as,Sn-X_	−41	−35 (−29)	−21	−15	−14
Δ*υ*_s,Sn-X_	−38	−32 (−29)	−19	−12	−10
Δ*δ*_C-H_	13	17 (19)	19	19	17

Available experimental results taken from Ref. [52] are given in parentheses

All values in cm^−1^

The negative values of Δ*υ*_as,Sn-X_ and Δ*υ*_s,Sn-X_ shown in Table 6 indicate that small red shifts are observed for the frequencies of Sn-X stretching vibrations. The occurrence of these red shifts is associated with a minor elongation of Sn-X bonds upon complexation (see Table S6). The elongation of Sn-X bonds and the accompanying red shifts of Sn-X stretching frequencies can be roughly explained by the charge transfers between SnX_2_ and C_6_H_6_. As it was discussed in the previous subsection, the SnX_2_ fragment of the complexes bears a slight negative charge and the plots of the electron density difference show regions of electron accumulation around the Sn and X atoms upon complexation. Such a charge transfer contributes to the red shifts. The NBO results reported in Ref. [20] confirm the existence of the charge transfer from the π-type orbitals of C_6_H_6_ to the empty orbitals of SnX_2_. Additionally, the charge transfer involving the π-type orbitals is reflected in the structural properties of the C_6_H_6_ ring. Our calculations reveal that the C-C bonds are elongated by ca. 0.002 Å upon the formation of SnX_2_ · · · C_6_H_6_. A region of growing *ρ* around the C atom adjacent to Sn has been clearly seen in Fig. 4. This implies that at the same time there is a reverse transfer toward C_6_H_6_, which also facilitates the red shifts. The NBO analysis has however established that the back-donation toward the π^*^-orbitals of C_6_H_6_ is negligible in comparison to the transfer toward the orbitals of SnX_2_ [20].

The calculated Δ*υ*_as,Sn-X_, Δ*υ*_s,Sn-X_ and Δ*δ*_C-H_ of **2** can be compared with the corresponding experimental data taken from Ref. [52]. The calculated values demonstrate good agreement with the experimental data. The reliability of the presented computational predictions of shifts in vibrational frequencies can be further proven through the inclusion of additional complexes in this study. Two additional complexes composed of SnF_2_ and chlorobenzene or toluene have been considered because their Δ*υ*_as,Sn-F_, Δ*υ*_s,Sn-F_ and Δ*δ*_C-H_ shifts were previously determined experimentally [52]. The results obtained for these two additional complexes are presented in detail in section S3. Suffice it to say here that the calculated shifts in the Sn-F and C-H vibrational frequencies of **2** and two additional complexes are in good agreement with experiment. Moreover, the calculated shifts of *υ*_as,Sn-F_ and *υ*_s,Sn-F_ reproduce the experimentally established trend in the magnitude of these shifts [52].

## Conclusions

In this work a variety of quantum-chemical methods have been used to provide an insight into the intermolecular interaction occurring in the complexes of SnX_2_ with C_6_H_6_. By analyzing the results of energy and electron density topology calculations, we conclude with the following remarks.

1. The complexes are rather weakly bound and the *E*_int_ energy between SnX_2_ and C_6_H_6_ turns out to be similar for all five complexes. A very small destabilizing effect associated with changes in the geometries of SnX_2_ and C_6_H_6_ appears during the formation of each complex. This effect does not exceed 4 % of the absolute values of *E*_int_.
2. The effects of electron correlation play a vital role in the proper description of the interaction in the five complexes. Among the DFT methods, those belonging to older DFT generations fail badly: both severe under- and overestimations of *E*_int_ are possible. The inclusion of the D3(BJ) correction improves their performance, but it tends to overestimate the role of long-range electron correlation. Newer density functionals without empirical dispersion correction (M06-2X and ωB97X) acquit themselves reasonably well.
3. The SAPT analysis reveals that the electrostatics is the dominant attractive component of *E*_int_ for all five complexes. However, the *E*_elst_ component is compensated by the *E*_exch_ component, and therefore, the stabilization of the complexes is determined to a great extent by the second-order component that is accountable for dispersion.
4. Based on the QTAIM and NCI results, the interaction between SnX_2_ and C_6_H_6_ can be classified as a closed-shell, noncovalent and attractive interaction. The formation of the complexes polarizes the electron density around the Sn atom toward the π-cloud of C_6_H_6_.
5. By integrating the electron density of the complexes over their QTAIM atomic basins, we have deduced a very small charge transfer from C_6_H_6_ to SnX_2_ for all five complexes.
6. The calculated shifts in the frequencies of Sn-X and C-H vibrations agree well with the available experimental data. The relatively small shifts of these vibrational frequencies upon complexation confirm that the interaction between SnX_2_ and C_6_H_6_ is rather weak and there is no appreciable change in the inner geometries of interacting SnX_2_ and C_6_H_6_.

## Electronic supplementary material

Below is the link to the electronic supplementary material.

ESM 1 (DOC 5.55 mb)

## References

[1] Haiduc I, Edelmann FT (1999). Supramolecular Organometallic Chemistry.

[2] Meyer F, Khan FA, Armentrout PB (1995). Thermochemistry of transition metal benzene complexes: Binding energies of M(C_6_H_6_)_x_^+^ (x = 1,2) for M = Ti to Cu. J Am Chem Soc.

[3] Kurikawa T, Takeda H, Hirano M, Judai K, Arita T, Nagao S, Nakajima A, Kaya K (1999) Electronic properties of organometallic metal-benzene complexes [M_n_(benzene)_m_ (M = Sc–Cu)]. Organometallics 18:1430–1438

[4] Han S, Singh NJ, Kang TY, Choi K-W, Choi S, Baek SJ, Kim KS, Kim SK (2010). Aromatic π–π interaction mediated by a metal atom: Structure and ionization of the bis(η^6^-benzene)chromium–benzene cluster. Phys Chem Chem Phys.

[5] Brunner H, Oescheya R, Nuber B (1996). Optically active transition metal complexes. Part 108. Synthesis, crystal structure and properties of a novel “quasi-meso” dinuclear η^6^-benzene-ruthenium(II) complex with chiral salicylaldiminato ligands. J Organomet Chem.

[6] Jiang J, Smith JR, Luo Y, Grennberg H, Ottosson H (2011). Multidecker bis(benzene)chromium: Opportunities for design of rigid and highly flexible molecular wires. J Phys Chem C.

[7] Haghiri A, Lerner H-W, Bats JW (2007). Tricarbonyl[η^6^-1-methyl-4-(trimethylsilyl)benzene]chromium(0). Acta Cryst E.

[8] Schmidbaur H, Schier A (2008). π-Complexation of post-transition metals by neutral aromatic hydrocarbons: The road from observations in the 19th century to new aspects of supramolecular chemistry. Organometallics.

[9] Haiduc I, Tiekink ERT, Zukerman-Schpector J, Gielen M, Davies AG, Pannell KH, Tiekink ERT (2008). Intermolecular tin · · · π-aryl interactions: Fact or artifact? A new bonding motif for supramolecular self-assembly in organotin compounds. Tin Chemistry: Fundamentals, Frontiers, and Applications.

[10] Neumann WP (1991). Germylenes and stannylenes. Chem Rev.

[11] Zabula AV, Hahn FE (2008). Mono- and bidentate benzannulated N-heterocyclic germylenes, stannylenes and plumbylenes. Eur J Inorg Chem.

[12] Mansell SM, Russell CA, Wass DF (2008). Synthesis and structural characterization of tin analogues of N-heterocyclic carbenes. Inorg Chem.

[13] Schäfer A, Winter F, Saak W, Haase D, Pöttgen R, Müller T (2011). Stannylium ions, a tin(II) arene complex, and a tin dication stabilized by weakly coordinating anions. Chem Eur J.

[14] Li J, Schenk C, Winter F, Scherer H, Trapp N, Higelin A, Keller S, Pöttgen R, Krossing I, Jones C (2012). Weak arene stabilization of bulky amido-germanium(II) and tin(II) monocations. Angew Chem Int Ed.

[15] Jeziorski B, Moszyński R, Szalewicz K (1994). Perturbation theory approach to intermolecular potential energy surfaces of van der Waals complexes. Chem Rev.

[16] Szalewicz K (2012). Symmetry-adapted perturbation theory of intermolecular forces. WIREs Comput Mol Sci.

[17] Bader RFW (1990). Atoms in Molecules: A Quantum Theory.

[18] Johnson ER, Keinan S, Mori-Sánchez P, Contreras-García J, Cohen AJ, Yang W (2010). Revealing noncovalent interactions. J Am Chem Soc.

[19] Matczak P, Wojtulewski S (2015). Performance of Møller-Plesset second-order perturbation theory and density functional theory in predicting the interaction between stannylenes and aromatic molecules. J Mol Model.

[20] Broeckaert L, Geerlings P, Růžička A, Willem R, De Proft F (2012). Can aromatic π-clouds complex divalent germanium and tin compounds? A DFT study. Organometallics.

[21] Chai J-D, Head-Gordon M (2008). Systematic optimization of long-range corrected hybrid density functionals. J Chem Phys.

[22] Dunning TH (1989). Gaussian basis sets for use in correlated molecular calculations. I. The atoms boron through neon and hydrogen. J Chem Phys.

[23] Peterson KA (2003). Systematically convergent basis sets with relativistic pseudopotentials. I. Correlation consistent basis sets for the post-d group 13–15 elements. J Chem Phys.

[24] Metz B, Stoll H, Dolg M (2000). Small-core multiconfiguration-Dirac–Hartree–Fock-adjusted pseudopotentials for post-d main group elements: Application to PbH and PbO. J Chem Phys.

[25] Slater JC (1974). The Self-Consistent Field for Molecular and Solids, Quantum Theory of Molecular and Solids.

[26] Vosko SH, Wilk L, Nusair M (1980). Accurate spin-dependent electron liquid correlation energies for local spin density calculations: A critical analysis. Can J Phys.

[27] Becke AD (1988). Density-functional exchange-energy approximation with correct asymptotic behavior. Phys Rev A.

[28] Lee C, Yang W, Parr RG (1988). Development of the Colle–Salvetti correlation-energy formula into a functional of the electron density. Phys Rev B.

[29] Becke AD (1993). Density-functional thermochemistry. III. The role of exact exchange. J Chem Phys.

[30] Zhao Y, Truhlar DG (2008). The M06 suite of density functionals for main group thermochemistry, thermochemical kinetics, noncovalent interactions, excited states, and transition elements: Two new functionals and systematic testing of four M06-class functionals and 12 other functionals. Theor Chem Acc.

[31] Hartree DR (1928). The wave mechanics of an atom with a non-Coulomb central field. Part I: Theory and methods. Proc Cambridge Philos Soc.

[32] Fock V (1930) Näherungsmethode zur Lösung des quantenmechanischen Mehrkörperproblems. Z Phys 61:126–148

[33] Møller C, Plesset MS (1934). Note on an approximation treatment for many-electron systems. Phys Rev.

[34] Grimme S (2003). Improved second-order Møller-Plesset perturbation theory by separate scaling of parallel- and antiparallel-spin pair correlation energies. J Chem Phys.

[35] Gauss J, Schleyer PR, Allinger NL, Clark T (1998). Coupled-cluster theory. Encyclopedia of Computational Chemistry.

[36] Boys SF, Bernardi F (1970). The calculation of small molecular interactions by the differences of separate total energies. Some procedures with reduced errors. Mol Phys.

[37] Burns LA, Marshall MS, Sherrill CD (2014). Comparing counterpoise-corrected, uncorrected, and averaged binding energies for benchmarking noncovalent interactions. J Chem Theory Comput.

[38] Frisch MJ, Trucks GW, Schlegel HB, Scuseria GE, Robb MA, Cheeseman JR, Scalmani G, Barone V, Mennucci B, Petersson GA, Nakatsuji H, Caricato M, Li X, Hratchian HP, Izmaylov AF, Bloino J, Zheng G, Sonnenberg JL, Hada M, Ehara M, Toyota K, Fukuda R, Hasegawa J, Ishida M, Nakajima T, Honda Y, Kitao O, Nakai H, Vreven T, Montgomery JA, Peralta JE, Ogliaro F, Bearpark M, Heyd JJ, Brothers E, Kudin KN, Staroverov VN, Keith T, Kobayashi R, Normand J, Raghavachari K, Rendell A, Burant JC, Iyengar SS, Tomasi J, Cossi M, Rega N, Millam JM, Klene M, Knox JE, Cross JB, Bakken V, Adamo C, Jaramillo J, Gomperts R, Stratmann RE, Yazyev O, Austin AJ, Cammi R, Pomelli C, Ochterski JW, Martin RL, Morokuma K, Zakrzewski VG, Voth GA, Salvador P, Dannenberg JJ, Dapprich S, Daniels AD, Farkas O, Foresman JB, Ortiz JV, Cioslowski J, Fox DJ (2013). Gaussian 09 D.01.

[39] Ahlrichs R, Armbruster MK, Bachorz RA, Bär M, Baron HP, Bauernschmitt R, Bischoff FA, Böcker S, Crawford N, Deglmann P, Della Sala F, Diedenhofen M, Ehrig M, Eichkorn K, Elliott S, Friese D, Furche F, Glöß A, Haase F, Häser M, Hättig C, Hellweg A, Höfener S, Horn H, Huber C, Huniar U, Kattannek M, Klopper W, Köhn A, Kölmel C, Kollwitz M, May K, Nava P, Ochsenfeld C, Öhm H, Pabst M, Patzelt H, Rappoport D, Rubner O, Schäfer A, Schneider U, Sierka M, Tew DP, Treutler O, Unterreiner B, von Arnim M, Weigend F, Weis P, Weiss H, Winter N (2014) TURBOMOLE 6.6. A development of University of Karlsruhe and Forschungszentrum Karlsruhe GmbH, 1989–2007, TURBOMOLE GmbH, since 2007, Karlsruhe, Germany, http://www.turbomole.com

[40] Williams HL, Chabalowski CF (2001). Using Kohn–Sham orbitals in symmetry-adapted perturbation theory to investigate intermolecular interactions. J Phys Chem A.

[41] Jansen G, Hesselmann A (2001). Comment on “Using Kohn − Sham orbitals in symmetry-adapted perturbation theory to investigate intermolecular interactions”. J Phys Chem A.

[42] Grüning M, Gritsenko OV, van Gisbergen SJA, Baerends EJ (2001). Shape corrections to exchange-correlation potentials by gradient-regulated seamless connection of model potentials for inner and outer region. J Chem Phys.

[43] Adamo C, Barone V (1999). Toward reliable density functional methods without adjustable parameters: The PBE0 model. J Chem Phys.

[44] Werner H-J, Knowles PJ, Knizia G, Manby FR, Schütz M, Celani P, Korona T, Lindh R, Mitrushenkov A, Rauhut G, Shamasundar KR, Adler TB, Amos RD, Bernhardsson A, Berning A, Cooper DL, Deegan MJO, Dobbyn AJ, Eckert F, Goll E, Hampel C, Hesselmann A, Hetzer G, Hrenar T, Jansen G, Köppl C, Liu Y, Lloyd AW, Mata RA, May AJ, McNicholas SJ, Meyer W, Mura ME, Nicklass A, O’Neill DP, Palmieri P, Peng D, Pflüger K, Pitzer R, Reiher M, Shiozaki T, Stoll H, Stone AJ, Tarroni R, Thorsteinsson T, Wang M (2012) MOLPRO 2012.1. University College Cardiff Consultants Limited, Cardiff, UK, http://www.molpro.net

[45] Werner H-J, Knowles PJ, Knizia G, Manby FR, Schütz M (2012). MOLPRO: A general-purpose quantum chemistry program package. WIREs Comput Mol Sci.

[46] Keith TA (2014). AIMAll (Version 14.11.23).

[47] Lu T, Chen F (2012). Multiwfn: A multifunctional wavefunction analyzer. J Comput Chem.

[48] Silvi B, Savin A (1994). Classification of chemical bonds based on topological analysis of electron localization functions. Nature.

[49] Jmol: An open-source Java viewer for chemical structures in 3D. http://www.jmol.org

[50] Humphrey W, Dalke A, Schulten K (1996). VMD – Visual Molecular Dynamics. J Mol Graphics.

[51] Alvarez S (2013). A cartography of the van der Waals territories. Dalton Trans.

[52] Boganov SE, Egorov MP, Nefedov OM (1999). Study of complexation between difluorostannylene and aromatics by matrix IR spectroscopy. Russ Chem Bull.

[53] Soteras I, Orozco M, Luque FJ (2008). Induction effects in metal cation–benzene complexes. Phys Chem Chem Phys.

[54] Khanmohammadi A, Raissi H, Mollania F, Hokmabadi L (2014). Molecular structure and bonding character of mono and divalent metal cations (Li^+^, Na^+^, K^+^, Be^2+^, Mg^2+^, and Ca^2+^) with substituted benzene derivatives: AIM, NBO, and NMR analyses. Struct Chem.

[55] Nziko VPN, Scheiner S (2015). S · · · π Chalcogen bonds between SF_2_ or SF_4_ and C − C multiple bonds. J Phys Chem A.

[56] Saberinasab M, Salehzadeh S, Maghsoud Y, Bayat M (2016). The significant effect of electron donating and electron withdrawing substituents on nature and strength of an intermolecular Se · · · π interaction. A theoretical study. Comput Theoret Chem.

[57] Kurth S, Perdew JP, Blaha P (1999). Molecular and solid-state tests of density functional approximations: LSD, GGAs, and meta-GGAs. Int J Quantum Chem.

[58] Grimme S, Ehrlich S, Goerigk L (2011). Effect of the damping function in dispersion corrected density functional theory. J Comput Chem.

[59] Schneebeli ST, Bochevarov AD, Friesner RA (2011). Parameterization of a B3LYP specific correction for noncovalent interactions and basis set superposition error on a gigantic data set of CCSD(T) quality noncovalent interaction energies. J Chem Theory Comput.

[60] van der Wijst T, Fonseca Guerra C, Swart M, Bickelhaupt FM, Lippert B (2009). A ditopic ion-pair receptor based on stacked nucleobase quartets. Angew Chem Int Ed.

[61] Fonseca Guerra C, van der Wijst T, Poater J, Swart M, Bickelhaupt FM (2010). Adenine versus guanine quartets in aqueous solution: Dispersion-corrected DFT study on the differences in π-stacking and hydrogen-bonding behavior. Theor Chem Acc.

[62] Zhang W, Truhlar DG, Tang M (2013). Tests of exchange-correlation functional approximations against reliable experimental data for average bond energies of 3d transition metal compounds. J Chem Theory Comput.

[63] Matczak P, Łukomska M (2014). Assessment of various density functionals for intermolecular N → Sn interactions: The test case of trimethyltin cyanide dimer. Comput Theoret Chem.

[64] Matczak P (2015). Assessment of various density functionals for intermolecular N → Sn interactions: The test case of poly(trimethyltin cyanide). Comput Theoret Chem.

[65] Zhao Y, Truhlar DG (2007). Density functionals for noncovalent interaction energies of biological importance. J Chem Theory Comput.

[66] Hohenstein EG, Sherrill CD (2012). Wavefunction methods for noncovalent interactions. WIREs Comput Mol Sci.

[67] Parker TM, Burns LA, Parrish RM, Ryno AG, Sherrill CD (2014). Levels of symmetry adapted perturbation theory (SAPT). I. Efficiency and performance for interaction energies. J Chem Phys.

[68] Cybulski SM, Chałasiński G, Moszyński R (1990). On decomposition of second-order Møller-Plesset supermolecular interaction energy and basis set effects. J Chem Phys.

[69] Cybulski SM, Lytle ML (2007). The origin of deficiency of the supermolecule second-order Møller-Plesset approach for evaluating interaction energies. J Chem Phys.

[70] Patkowski K, Szalewicz K (2007). Frozen core and effective core potentials in symmetry-adapted perturbation theory. J Chem Phys.

[71] Riley KE, Ford CL, Demouchet K (2015). Comparison of hydrogen bonds, halogen bonds, C-H · · · π interactions, and C-X · · · π interactions using high-level ab initio methods. Chem Phys Lett.

[72] Kalugina YN, Thakkar AJ (2015). Electric properties of stannous and stannic halides: How good are the experimental values?. Chem Phys Lett.

[73] Bader RFW (1998). A bond path: A universal indicator of bonded interactions. J Phys Chem A.

[74] Espinosa E, Alkorta I, Elguero J, Molins E (2002). From weak to strong interactions: A comprehensive analysis of the topological and energetic properties of the electron density distribution involving X–H · · · F–Y systems. J Chem Phys.

[75] Grabowski SJ, Leszczynski J (2009). The enhancement of X–H · · · π hydrogen bond by cooperativity effects — Ab initio and QTAIM calculations. Chem Phys.

[76] Bianchi R, Gervasio G, Marabello D (2000). Experimental electron density analysis of Mn_2_(CO)_10_: Metal-metal and metal-ligand bond characterization. Inorg Chem.

[77] Van der Maelen JF, Cabeza JA (2012). QTAIM analysis of the bonding in Mo − Mo bonded dimolybdenum complexes. Inorg Chem.

[78] Cukrowski I, de Lange JH, Mitoraj M (2014). Physical nature of interactions in Zn^II^ complexes with 2,2′-bipyridyl: Quantum theory of atoms in molecules (QTAIM), interacting quantum atoms (IQA), noncovalent interactions (NCI), and extended transition state coupled with natural orbitals for chemical valence (ETS-NOCV) comparative studies. J Phys Chem A.

