# Supplementary material for: Theoretical insight into the interaction between SnX2 (X = H, F, Cl, Br, I) and benzene
Source: J Mol Model. 2016 Aug 15;22(9):208. doi: 10.1007/s00894-016-3053-6 (PMC4985535; doi:10.1007/s00894-016-3053-6)
Supplement: Supplementary file 1 — (DOC 5.55 mb) [file 894_2016_3053_MOESM1_ESM.doc]

**Electronic Supplementary Material**

for

Theoretical insight into the interaction between SnX_2_ (X = H, F, Cl, Br, I) and benzene

by

Piotr Matczak

Department of Theoretical and Structural Chemistry, Faculty of Chemistry, University of Łódź, Pomorska 163/165, 90-236 Lodz, Poland

|  |  |  |
| --- | --- | --- |

**S1. Various modes of the interaction between SnX_2_ and C_6_H_6_**

In this work, the complexes **1**–**5** adopt the geometries that exhibit three common features: the Sn atom of SnX_2_ sits nearly on one of the C atoms of C_6_H_6_; the X atoms of SnX_2_ are directed outward the C_6_H_6_ ring; the molecular planes of SnX_2_ and C_6_H_6_ are approximately parallel. These geometries result from the investigated mode of intermolecular interaction that involves the formally empty *p*-orbital on the Sn atom and the *π*-cloud of C_6_H_6_. It is necessary to justify our investigation of the complexes in these geometries because such an interaction mode might also lead to other stable geometries of the complexes. Besides, some other modes of the intermolecular interaction between SnX_2_ and C_6_H_6_ might occur for **1**–**5**. Such other modes would obviously be manifested by different molecular geometries of the complexes.

In order to resolve the aforementioned issue, we have performed a detailed investigation of the potential energy surface (PES) of each complex. The calculations carried out at the ωB97X/aug-cc-pVTZ(-PP) level of theory yield several stationary points on the PES of each complex. The analysis of harmonic vibrational frequencies confirms that these stationary points are local energy minima. The molecular structures corresponding to these local energy minima are presented schematically in Fig. S1. The interaction modes marked **A** and **C** have been detected for all five complexes, while the remaining modes are observed only for a part of complexes. Table S1 presents the differences between the ωB97X/aug-cc-pVTZ(-PP) total energies of structures detected for each complex. The differences are calculated relative to the total energy of the preferred structure, that is, the one with the lowest total energy. It is evident from what Table S1 shows that mode **A** is most energetically favorable, while modes **B**–**F** are less stable (their relative energies adopt positive values). The mode involving the interaction between the formally empty *p*-orbital of Sn and the *π*-cloud is responsible for two geometrical arrangements of SnX_2_···C_6_H_6_. They are marked **A** and **B** in Fig. S1. The former turns out to be more energetically favorable than the latter. This is also confirmed by the *E*_complex_ and *E*_int_ values listed in Table S2. These values have been calculated at the ωB97X/aug-cc-pVTZ(-PP) level of theory. Structures **C**–**F** display quite different interaction modes than those of **A** and **B**. They may probably be ascribed to either hydrogen bonding (**E** and **F**) or halogen bonding (**C** and **D** for SnX_2_ being tin(II) dihalides).

We conclude that the results of PES exploration for **1**–**5** prove the energetic preference of the mode involving the interaction between the formally empty *p*-orbital of Sn and the *π*-cloud of C_6_H_6_. Moreover, structures **A** are the global energy minima of **1**–**5**, and therefore, we focus on them in the present work.

**
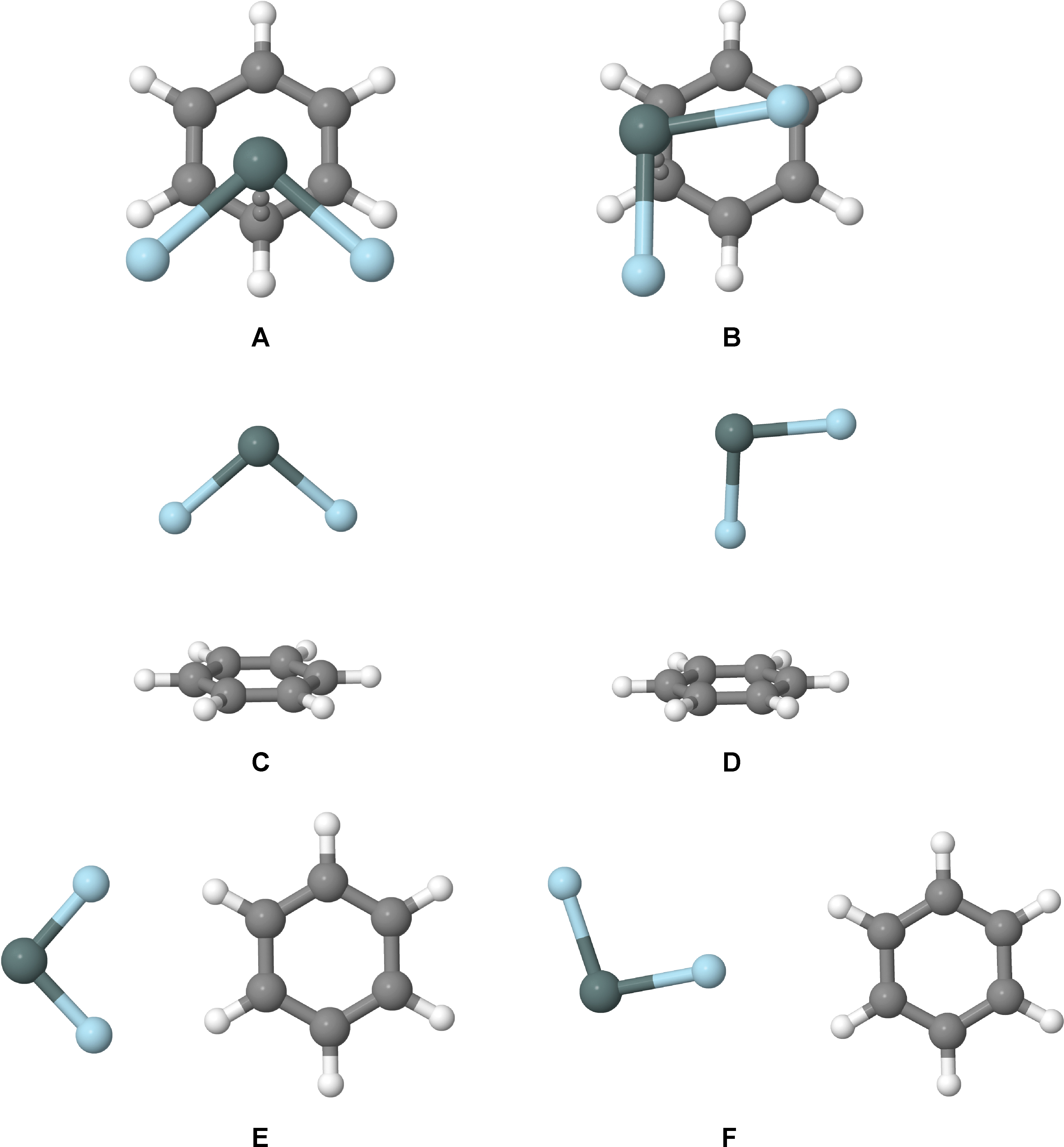
**

**Fig. S1** Various energy minima on the PES’s of SnX_2_···C_6_H_6_

**Table S1** Relative energies between the total energies of each complex in its various geometries (structures **A**–**F** from Fig. S1). The corresponding relative energies between the total energies including the correction for the unscaled zero-point vibrational energy are given in parentheses

| Structure | Complex | | | | |
| --- | --- | --- | --- | --- | --- |
|  | **1** | **2** | **3** | **4** | **5** |
| **A** | 0.0  (0.0) | 0.0  (0.0) | 0.0  (0.0) | 0.0  (0.0) | 0.0  (0.0) |
| **B** | 1.1  (1.0) | - | 1.7  (1.6) | 1.6  (1.5) | 1.3  (1.3) |
| **C** | 8.2  (7.0) | 9.8  (9.3) | 8.7  (8.2) | 8.1  (7.6) | 7.4  (6.9) |
| **D** | - | - | 8.8  (8.5) | 8.1  (7.7) | 7.0  (6.6) |
| **E** | - | 7.1  (6.9) | - | - | - |
| **F** | - | - | 8.3  (7.9) | - | - |

All values in kcal/mol

**Table S2** Complexation energies and interaction energies (in parentheses) calculated for five complexes in their various geometries (structures **A**–**F** from Fig. S1)

| Structure | Complex | | | | |
| --- | --- | --- | --- | --- | --- |
|  | **1** | **2** | **3** | **4** | **5** |
| **A** | -7.6  (-9.3) | -8.8  (-9.7) | -8.8  (-9.7) | -8.5  (-9.5) | -8.2  (-9.1) |
| **B** | -6.6  (-8.3) | - | -7.2  (-7.9) | -7.1  (-7.9) | -6.9  (-7.7) |
| **C** | -0.7  (-1.1) | 0.3  (0.1) | -0.7  (-0.8) | -1.0  (-1.2) | -1.4  (-1.5) |
| **D** | - | - | -0.4  (-0.7) | -1.0  (-1.2) | -1.7  (-1.9) |
| **E** | - | -2.0  (-2.4) | - | - | - |
| **F** | - | - | -1.0  (-1.2) | - | - |

All values in kcal/mol

**S2. Calculations of the CCSD(T) interaction energy extrapolated to the CBS limit**

The coupled-cluster method incorporating single, double and perturbative triple excitations (CCSD(T)), together with an extrapolation to the complete basis set (CBS) limit, is capable of delivering very accurate interaction energies for various molecular complexes [1]. Thus, the CCSD(T)/CBS level of theory is used in this work to provide the most accurate estimation of the interaction energy *E*_int_ between SnX_2_ and C_6_H_6_ in **1**–**5**. However, the CCSD(T)/CBS calculations are very time-consuming, especially with larger basis sets employed for extrapolating to the CBS limit. In order to decrease the computational cost of our CCSD(T)/CBS calculations, a composite scheme is used to obtain the CCSD(T)/CBS total energies of SnX_2_, C_6_H_6_ and SnX_2_···C_6_H_6_. Subsequently, the *E*_int_ energies at the CBS limit are determined using these total energies. Within the composite scheme, each CCSD(T)/CBS total energy $E_{\text{tot}}^{\text{CCSD(T)/CBS}}$ is approximated by the following formula:

$E_{\text{tot}}^{\text{CCSD(T)/CBS}}$ = $E_{\text{tot}}^{\text{HF/CBS}}$ + $E_{\text{corr}}^{\text{MP2/CBS}}$ + *Δ*^CCSD(T)^ (S1)

where $E_{\text{tot}}^{\text{HF/CBS}}$ is the Hartree-Fock total energy at the CBS limit, $E_{\text{corr}}^{\text{MP2/CBS}}$ is the correlation energy yielded by the second-order Møller-Plesset perturbation theory at the CBS limit, and *Δ*^CCSD(T)^ is the CCSD(T) correction for the correlation energy. $E_{\text{tot}}^{\text{HF/CBS}}$ is estimated by the three-point extrapolation proposed by Feller [2]. This extrapolation is based on a series of three calculations with successively increasing basis sets, from aug-cc-pVTZ(-PP) to aug-cc-pVQZ(-PP) and to aug-cc-pV5Z(-PP) in our case [3]. The resulting energies $E_{\text{tot}}^{\text{HF/aug-cc-pVTZ(-PP)}}$, $E_{\text{tot}}^{\text{HF/aug-cc-pVQZ(-PP)}}$ and $E_{\text{tot}}^{\text{HF/aug-cc-pV5Z(-PP)}}$ are inserted into the exponential expression

$E_{\text{tot}}^{\text{HF/aug-cc-pVXZ(-PP)}}$ = $E_{\text{tot}}^{\text{HF/CBS}}$ + A · exp(–*B* · *X*) (S2)

and the least-squares fitting procedure of the $E_{\text{tot}}^{\text{HF/CBS}}$, *A* and *B* coefficients allows us to achieve the best fit of $E_{\text{tot}}^{\text{HF/CBS}}$. The symbol *X* stands for the cardinal number of Dunning’s basis set employed (i.e., three for aug-cc-pVTZ(-PP), four for aug-cc-pVQZ(-PP), and five for aug-cc-pV5Z(-PP)). $E_{\text{corr}}^{\text{MP2/CBS}}$ is estimated using the two-point extrapolation proposed by Halkier et al [4]. This extrapolation relies on the correlation energies calculated at the MP2/aug-cc-pVQZ(-PP) and MP2/aug-cc-pV5Z(-PP) levels of theory. The resulting energies $E_{\text{corr}}^{\text{MP2/aug-cc-pVQZ(-PP)}}$ and $E_{\text{corr}}^{\text{MP2/aug-cc-pV5Z(-PP)}}$ are inserted into the following formula.

$E_{\text{corr}}^{\text{MP2/CBS}}$ = $\frac{5^{3}}{5^{3}-4^{3}}E_{\text{corr}}^{\text{MP2/aug}-\text{cc}-\text{pV5Z(-PP)}}-\frac{4^{3}}{5^{3}-4^{3}}E_{\text{corr}}^{\text{MP2/aug}-\text{cc}-\text{pVQZ(-PP)}}$ (S3)

Because the CCSD(T)/aug-cc-pV5Z(-PP) calculations turned out to be prohibitively expensive for **1**–**5**, the sum of $E_{\text{tot}}^{\text{HF/CBS}}$ and $E_{\text{corr}}^{\text{MP2/CBS}}$ is improved by the CCSD(T) correction term *Δ*^CCSD(T)^ [1]. This term is determined as a difference between the CCSD(T) and MP2 correlation energies calculated using the smaller, computationally tractable basis set, namely aug-cc-pVQZ(-PP).

*Δ*^CCSD(T)^ = $E_{\text{corr}}^{\text{CCSD(T)/aug-cc-pVQZ(-PP)}}$ – $E_{\text{corr}}^{\text{MP2/aug-cc-pVQZ(-PP)}}$ (S4)

The $E_{\text{tot}}^{\text{HF/aug-cc-pVXZ(-PP)}}$, $E_{\text{corr}}^{\text{MP2/aug-cc-pVXZ(-PP)}}$ and $E_{\text{corr}}^{\text{CCSD(T)/aug-cc-pVXZ(-PP)}}$ energies used in the extrapolation scheme are counterpoise-corrected energies [5].

**S3. Results of calculations for additional complexes**

Two additional complexes, namely the complex of SnF_2_ with chlorobenzene (**6**) and the complex of SnF_2_ with toluene (**7**), have been investigated here in order to find out to what extent our theoretical computations mimic the experimental trend in the shift of Sn-F stretching frequencies [6]. The PES’s of **6** and **7** have been explored at the ωB97X/aug-cc-pVTZ(-PP) level of theory. This level has been used to perform both the geometry optimization and harmonic vibrational frequency analysis of the two complexes. Several energy minima on the PES’s have been found and the structures that correspond to these minima are depicted in Figs. S2 and S3. Table S3 presents the relative energies of the structures shown in Figs. S2 and S3. Structure **G** turns out to be most energetically favorable for **6**, whereas structure **I** is preferred for **7**. Both structures exhibit an arrangement of the SnF_2_ and aromatic ring fragments in which the resulting intermolecular interaction mode involves the interaction between the formally empty *p*-orbital of Sn and the *π*-cloud of the chlorobenzene or toluene molecule. As evidenced by the values of interaction energy in Table S4, the interaction between SnF_2_ and the aromatic molecules is also stronger for the two structures than for the remaining structures.

The shifts in the theoretical and experimental frequencies of Sn-F and C-H vibrations for **6** and **7** are listed in Table S5. Our computational results are in reasonable agreement with experiment. If the shifts of calculated Sn-F stretching frequencies in a series containing **2**, **6** and **7** are taken into consideration, it is seen that the magnitude of these shifts grows in the order **6** < **2** < **7**. Such a trend was previously established on the basis of experimental measurements [6], and the results of our calculations reproduce this trend. Moreover, the values of the complexation and interaction energies of **2**, **6** and **7** (Tables S2 and S4) confirm the previously proposed hypothesis [6] that the increase in the shift of Sn-F stretching frequencies upon complexation in the series **6** < **2** < **7** is in agreement with the increase in the stability of these complexes.


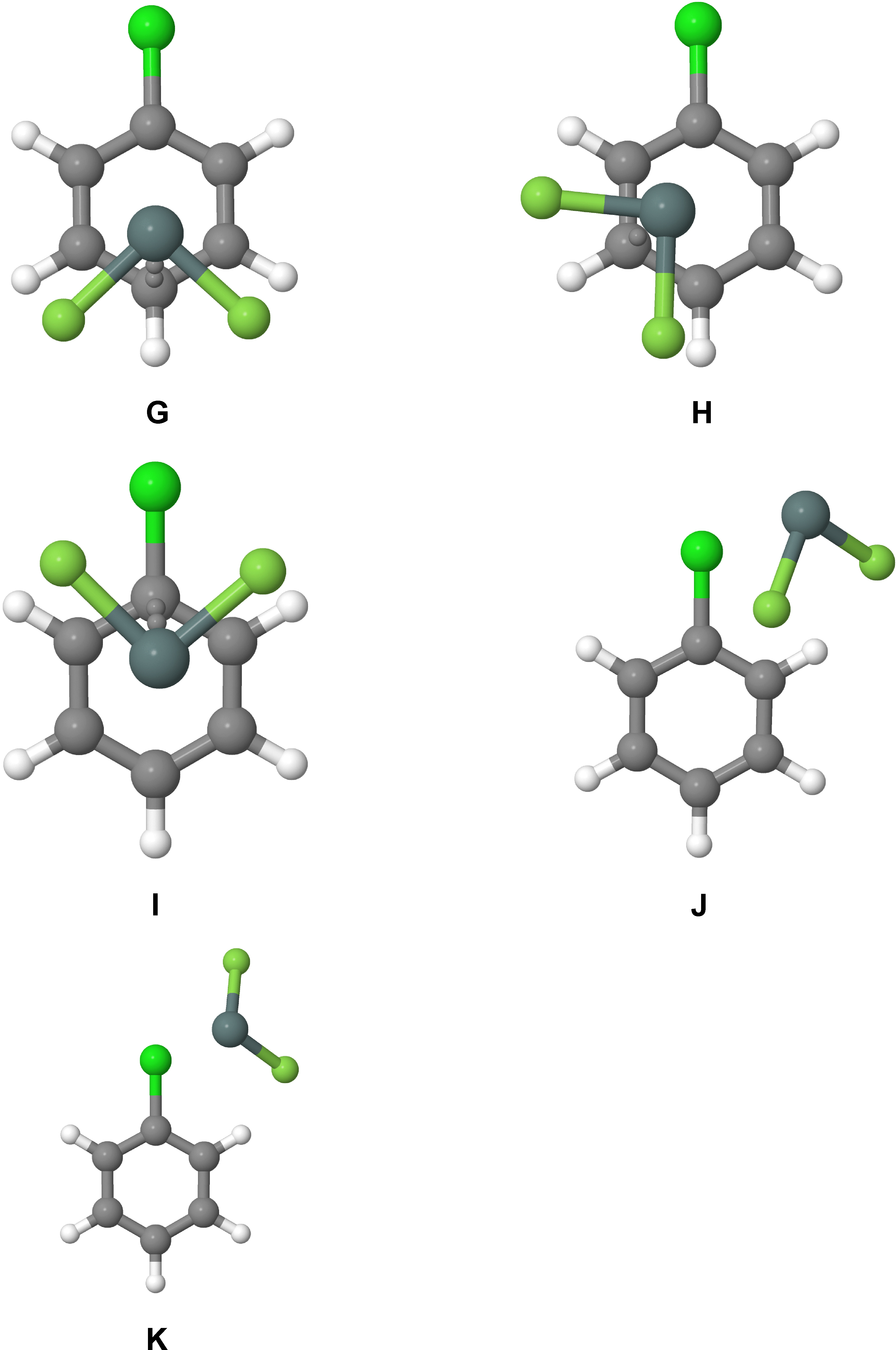


**Fig. S2** Various energy minima on the PES of **6**


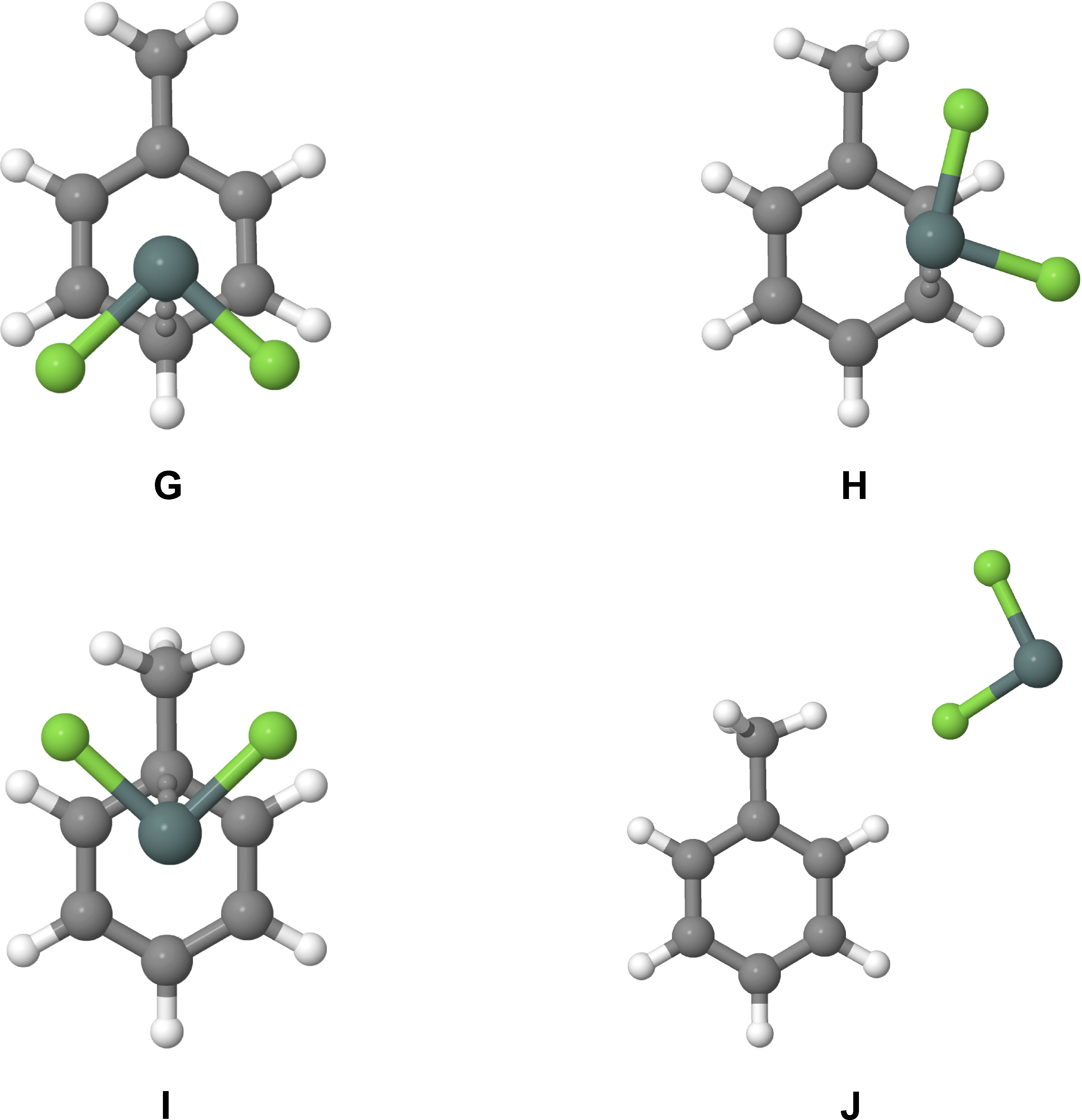


**Fig. S3** Various energy minima on the PES of **7**

**Table S3** Relative energies between the total energies of each complex in its various geometries (structures **G**–**K** in Figs. S2 and S3). The corresponding relative energies between the total energies including the correction for the unscaled zero-point vibrational energy are given in parentheses

| Structure | Complex | |
| --- | --- | --- |
|  | **6** | **7** |
| **G** | 0.0  (0.0) | 1.4  (1.2) |
| **H** | 0.2  (0.2) | 0.1  (0.1) |
| **I** | 1.9  (1.9) | 0.0  (0.0) |
| **J** | 0.4  (0.4) | 9.6  (9.1) |
| **K** | 0.7  (0.7) | - |

All values in kcal/mol

**Table S4** Complexation energies and interaction energies (in parentheses) calculated for two complexes in their various geometries (structures **G**–**K** in Figs. S2 and S3)

| Structure | Complex | |
| --- | --- | --- |
|  | **6** | **7** |
| **G** | -8.0  (-8.6) | -9.3  (-10.2) |
| **H** | -7.8  (-8.4) | -10.3  (-11.5) |
| **I** | -6.1  (-6.7) | -10.4  (-11.6) |
| **J** | -7.6  (-8.4) | -1.5  (-1.9) |
| **K** | -7.4  (-8.0) | - |

All values in kcal/mol

**Table S5** Shifts in vibrational frequencies of **6** and **7** as the result of complex formation. Experimental results taken from Ref. [6] are given in parentheses

| Frequency shift | Complex | |
| --- | --- | --- |
|  | **6** | **7** |
| Δ*υ*_as,Sn-F_ | -32  (-22) | -42  (-35) |
| Δ*υ*_s,Sn-F_ | -29  (-27) | -38  (-30) |
| Δ*δ*_C-H_ | 17  (21) | 9  (9) |

All values in cm^-1^

**S4. Additional tables with results**

**Table S6** Geometrical parameters (*d*_Sn···C_, *a*_Sn···C–H_, *d*_Sn–X_, *a*_X–Sn–X_) for **1**–**5** optimized at the ωB97X/aug-cc-pVTZ(-PP) level of theory. Changes in the *d*_Sn–X_ distance (Δ*d*_Sn–X_) and the *a*_X–Sn–X_ angle (Δ*a*_X–Sn–X_) upon complexation are also shown

| Parameter | Complex | | | | |
| --- | --- | --- | --- | --- | --- |
|  | **1** | **2** | **3** | **4** | **5** |
| *d*_Sn···C_ | 2.919 | 2.998 | 3.029 | 3.050 | 3.076 |
| *a*_Sn···C–H_ | 88.2 | 95.9 | 95.8 | 96.2 | 96.2 |
| *d*_Sn–X_ | 1.775 | 1.964 | 2.396 | 2.551 | 2.765 |
| *a*_X–Sn–X_ | 90.7 | 94.5 | 96.7 | 97.6 | 98.6 |
| Δ*d*_Sn–X_ ^a^ | 0.007 | 0.017 | 0.029 | 0.034 | 0.037 |
| Δ*a*_X–Sn–X_ ^b^ | -0.2 | -1.2 | -1.3 | -1.3 | -1.4 |

Distances in Å, angles in º

^a^ Calculated by subtracting the *d*_Sn–X_ value of the isolated SnX_2_ molecule from the corresponding value found for the SnX_2_···C_6_H_6_ complexes

^b^ Calculated by subtracting the *a*_X–Sn–X_ value of the isolated SnX_2_ molecule from the corresponding value found for the SnX_2_···C_6_H_6_ complexes

**Table S7** Uncorrected interaction energies calculated using various methods. The corresponding basis set superposition errors estimated by the full counterpoise method are presented in parentheses

| Method | Complex | | | | |
| --- | --- | --- | --- | --- | --- |
|  | **1** | **2** | **3** | **4** | **5** |
| HF | -2.2  (0.1) | -4.9  (0.4) | -3.2  (0.3) | -2.8  (0.2) | -2.2  (0.1) |
| SVWN | -14.7  (0.2) | -13.7  (0.4) | -13.4  (0.3) | -13.2  (0.4) | -12.4  (0.2) |
| BLYP | -3.5  (0.2) | -2.3  (0.4) | -1.2  (0.3) | -0.7  (0.3) | 0.0  (0.2) |
| BLYP-D3(BJ) | -11.4  (0.0) | -11.0  (0.2) | -11.7  (0.1) | -11.8  (0.2) | -11.6  (0.1) |
| B3LYP | -4.7  (0.1) | -4.6  (0.4) | -3.5  (0.3) | -3.1  (0.3) | -2.4  (0.2) |
| B3LYP-D3(BJ) | -11.3  (0.0) | -11.9  (0.2) | -12.3  (0.1) | -12.4  (0.1) | -12.1  (0.1) |
| M06-2X | -10.6  (0.2) | -12.1  (0.4) | -11.5  (0.4) | -11.3  (0.3) | -11.0  (0.2) |
| ωB97X | -9.5  (0.2) | -10.0  (0.3) | -10.0  (0.3) | -9.8  (0.3) | -9.4  (0.3) |
| MP2 | -14.8  (3.5) | -15.1  (4.1) | -16.6  (4.3) | -16.7  (4.2) | -18.4  (5.8) |
| SCS-MP2 | -12.2  (3.3) | -12.9  (3.9) | -13.9  (4.1) | -13.9  (4.1) | -15.3  (5.5) |
| CCSD | -10.8  (3.2) | -12.5  (3.8) | -12.4  (3.9) | -12.2  (3.9) | -13.4  (5.4) |
| CCSD(T) | -12.3  (3.3) | -13.6  (4.0) | -13.8  (4.1) | -13.7  (4.1) | -15.0  (5.6) |

All values in kcal/mol

**Table S8** Mean signed errors of uncorrected, full and “half-half” counterpoise-corrected interaction energies of **1**–**5** calculated by MP2, SCS-MP2, CCSD and CCSD(T) relative to the CCSD(T)/CBS results

| Method | *E*_int_ ^a^ | | |
| --- | --- | --- | --- |
|  | uncorrected | full | half-half |
| MP2 | -5.46 | -1.07 | -3.27 |
| SCS-MP2 | -2.78 | 1.39 | -0.70 |
| CCSD | -1.37 | 2.65 | 0.64 |
| CCSD(T) | -2.82 | 1.40 | -0.71 |

All values in kcal/mol

^a^ Positive values correspond to an underestimation of the strength of the interaction in **1**–**5** with respect to the CCSD(T)/CBS results, while negative values mean overestimation

**Table S9** Cartesian coordinates for **1**–**5**

| Complex/Atom | Cartesian coordinate | | |
| --- | --- | --- | --- |
|  | X | Y | Z |
| **1** | | | |
| C | -1.598763 | 0.200929 | 1.205529 |
| C | -1.606291 | -0.497376 | 0.000000 |
| C | -1.598763 | 0.200929 | -1.205529 |
| C | -1.598763 | 1.588180 | -1.203125 |
| C | -1.602806 | 2.279015 | 0.000000 |
| C | -1.598763 | 1.588180 | 1.203125 |
| H | -1.594874 | -0.344037 | 2.140720 |
| H | -1.638157 | -1.579940 | 0.000000 |
| H | -1.594874 | -0.344037 | -2.140720 |
| H | -1.592102 | 2.130269 | -2.139616 |
| H | -1.599940 | 3.361408 | 0.000000 |
| H | -1.592102 | 2.130269 | 2.139616 |
| Sn | 1.306902 | -0.675490 | 0.000000 |
| H | 0.945915 | -1.869301 | 1.263601 |
| H | 0.945915 | -1.869301 | -1.263601 |
| **2** | | | |
| C | -1.099422 | 0.745272 | -0.118373 |
| C | -1.350072 | -0.568775 | -0.491137 |
| C | -0.311522 | -1.489194 | -0.531287 |
| C | 0.978917 | -1.099825 | -0.198713 |
| C | 1.233551 | 0.213189 | 0.174745 |
| C | 0.193960 | 1.138209 | 0.214433 |
| H | -1.907181 | 1.463350 | -0.068536 |
| H | -2.355970 | -0.875082 | -0.747382 |
| H | -0.508943 | -2.513656 | -0.819653 |
| H | 1.786600 | -1.819642 | -0.227583 |
| H | 2.233975 | 0.519214 | 0.450717 |
| H | 0.389040 | 2.159408 | 0.515861 |
| Sn | -0.396527 | 0.118836 | 2.969257 |
| F | -1.545265 | 1.711873 | 3.004095 |
| F | 1.250595 | 1.109886 | 3.373554 |
| **3** | | | |
| C | -1.312947 | 0.776395 | -0.499239 |
| C | -1.351952 | -0.601382 | -0.333232 |
| C | -0.190633 | -1.296221 | -0.023643 |
| C | 1.012350 | -0.609533 | 0.120519 |
| C | 1.048826 | 0.772395 | -0.047885 |
| C | -0.114865 | 1.463294 | -0.357354 |
| H | -2.219709 | 1.316956 | -0.738370 |
| H | -2.287349 | -1.134423 | -0.442962 |
| H | -0.216883 | -2.368714 | 0.118435 |
| H | 1.919511 | -1.150303 | 0.360512 |
| H | 1.983529 | 1.303692 | 0.075129 |
| H | -0.087235 | 2.537469 | -0.485948 |
| Sn | 0.190909 | -0.082811 | 2.987813 |
| Cl | 2.435497 | 0.667055 | 3.364181 |
| Cl | 0.599766 | -2.407247 | 3.402045 |
| **4** | | | |
| C | -1.313433 | 0.753903 | -0.542283 |
| C | -1.337979 | -0.622488 | -0.362756 |
| C | -0.172728 | -1.299712 | -0.030046 |
| C | 1.019562 | -0.596731 | 0.124143 |
| C | 1.041585 | 0.783741 | -0.058459 |
| C | -0.125862 | 1.456984 | -0.391215 |
| H | -2.223291 | 1.280768 | -0.799519 |
| H | -2.265083 | -1.168212 | -0.480128 |
| H | -0.187960 | -2.371237 | 0.121132 |
| H | 1.930492 | -1.124148 | 0.379599 |
| H | 1.968130 | 1.327907 | 0.070586 |
| H | -0.109475 | 2.529991 | -0.530895 |
| Sn | 0.141330 | -0.048941 | 2.990836 |
| Br | 2.498487 | 0.795140 | 3.481750 |
| Br | 0.545037 | -2.510344 | 3.527255 |
| **5** | | | |
| C | -1.322792 | 0.757288 | -0.565271 |
| C | -1.336901 | -0.621253 | -0.401780 |
| C | -0.168616 | -1.292156 | -0.067667 |
| C | 1.016291 | -0.580888 | 0.104191 |
| C | 1.028008 | 0.801558 | -0.063381 |
| C | -0.142466 | 1.468621 | -0.397518 |
| H | -2.235179 | 1.279321 | -0.823397 |
| H | -2.258275 | -1.173530 | -0.532795 |
| H | -0.175753 | -2.365832 | 0.068901 |
| H | 1.930533 | -1.103973 | 0.357296 |
| H | 1.949242 | 1.352231 | 0.076517 |
| H | -0.134012 | 2.543190 | -0.525324 |
| Sn | 0.110249 | -0.068386 | 2.998536 |
| I | 2.613398 | 0.916189 | 3.638667 |
| I | 0.535087 | -2.725758 | 3.633027 |

All values in Å

**Table S10** Cartesian coordinates for **6** and **7**

| Complex/Atom | Cartesian coordinate | | |
| --- | --- | --- | --- |
|  | X | Y | Z |
| **6** | | | |
| C | -1.273315 | 0.736801 | -0.413140 |
| C | -1.301887 | -0.642569 | -0.277316 |
| C | -0.122591 | -1.318621 | 0.000623 |
| C | 1.071990 | -0.619593 | 0.141560 |
| C | 1.081766 | 0.764680 | 0.002959 |
| C | -0.092366 | 1.449676 | -0.274950 |
| Cl | -2.743697 | 1.587281 | -0.757881 |
| H | -2.236486 | -1.175075 | -0.386642 |
| H | -0.135172 | -2.393207 | 0.124612 |
| H | 1.987495 | -1.149175 | 0.369585 |
| H | 2.006587 | 1.311607 | 0.128817 |
| H | -0.097175 | 2.525500 | -0.382523 |
| Sn | 0.319736 | -0.187267 | 3.035959 |
| F | 2.192381 | 0.397720 | 3.095768 |
| F | 0.751548 | -2.101134 | 3.092568 |
| **7** | | | |
| C | -1.229264 | 0.787023 | -0.040483 |
| C | -1.067179 | -0.598890 | 0.034846 |
| C | 0.221930 | -1.116728 | -0.115446 |
| C | 1.305542 | -0.282470 | -0.356482 |
| C | 1.123723 | 1.090072 | -0.444587 |
| C | -0.146959 | 1.622773 | -0.281360 |
| H | -2.216644 | 1.209204 | 0.102052 |
| H | 0.371769 | -2.186280 | -0.031480 |
| H | 2.294484 | -0.707648 | -0.470457 |
| H | 1.968030 | 1.741063 | -0.629881 |
| H | -0.298052 | 2.693252 | -0.336348 |
| C | -2.245491 | -1.505592 | 0.255491 |
| H | -2.681885 | -1.795170 | -0.702269 |
| H | -1.945657 | -2.409266 | 0.784659 |
| H | -3.012365 | -1.007315 | 0.847246 |
| Sn | -0.229763 | -0.082629 | 2.869906 |
| F | -0.407907 | -2.040253 | 2.960274 |
| F | -2.163162 | 0.234294 | 3.054318 |

All values in Å

**References**

1. Riley KE, Pitoňák M, Jurečka P, Hobza P (2010) Stabilization and structure calculations for noncovalent interactions in extended molecular systems based on wave function and density functional theories. Chem Rev 110:5023–5063

2. Feller D (1993) The use of systematic sequences of wave functions for estimating the complete basis set, full configuration interaction limit in water. J Chem Phys 98:7059–7071

3. Peterson KA (2003) Systematically convergent basis sets with relativistic pseudopotentials. I. Correlation consistent basis sets for the post-d group 13–15 elements. J Chem Phys 119:11099–11112

4. Halkier A, Helgaker T, Jørgensen P, Klopper W, Koch H, Olsen J, Wilson AK (1998) Basis-set convergence in correlated calculations on Ne, N_2_, and H_2_O. Chem Phys Lett 286:243–252

5. Boys SF, Bernardi F (1970) The calculation of small molecular interactions by the differences of separate total energies. Some procedures with reduced errors. Mol Phys 19:553–566

6. Boganov SE, Egorov MP, Nefedov OM (1999) Study of complexation between difluorostannylene and aromatics by matrix IR spectroscopy. Russ Chem Bull 48:98–103
